# Comparative Analysis of Secondary Metabolites Produced by *Ascochyta fabae* under In Vitro Conditions and Their Phytotoxicity on the Primary Host, *Vicia faba*, and Related Legume Crops

**DOI:** 10.3390/toxins15120693

**Published:** 2023-12-09

**Authors:** Eleonora Barilli, Pierluigi Reveglia, Francisco J. Agudo-Jurado, Vanessa Cañete García, Alessio Cimmino, Antonio Evidente, Diego Rubiales

**Affiliations:** 1 Institute for Sustainable Agriculture, Spanish National Research Council (CSIC), 14004 Córdoba, Spain; preveglia@ias.csic.es (P.R.); q92agjuf@uco.es (F.J.A.-J.); q22cagav@uco.es (V.C.G.); 2 Department of Chemical Science, University of Naples Federico II (UNINA), 80126 Naples, Italy; alessio.cimmino@unina.it (A.C.); evidente@unina.it (A.E.); 3 Institute of Sciences of Food Production, National Research Council, 70126 Bari, Italy

**Keywords:** fungal metabolites, Ascochyta blight, legumes, phytotoxins

## Abstract

Ascochyta blight, caused by *Ascochyta fabae*, poses a significant threat to faba bean and other legumes worldwide. Necrotic lesions on stems, leaves, and pods characterize the disease. Given the economic impact of this pathogen and the potential involvement of secondary metabolites in symptom development, a study was conducted to investigate the fungus’s ability to produce bioactive metabolites that might contribute to its pathogenicity. For this investigation, the fungus was cultured in three substrates (Czapek-Dox, PDB, and rice). The produced metabolites were analyzed by NMR and LC-HRMS methods, resulting in the dereplication of seven metabolites, which varied with the cultural substrates. Ascochlorin, ascofuranol, and (*R*)-mevalonolactone were isolated from the Czapek-Dox extract; ascosalipyrone, benzoic acid, and tyrosol from the PDB extract; and ascosalitoxin and ascosalipyrone from the rice extract. The phytotoxicity of the pure metabolites was assessed at different concentrations on their primary hosts and related legumes. The fungal exudates displayed varying degrees of phytotoxicity, with the Czapek-Dox medium’s exudate exhibiting the highest activity across almost all legumes tested. The species belonging to the genus *Vicia* spp. were the most susceptible, with faba bean being susceptible to all metabolites, at least at the highest concentration tested, as expected. In particular, ascosalitoxin and benzoic acid were the most phytotoxic in the tested condition and, as a consequence, expected to play an important role on necrosis’s appearance.

## 1. Introduction

Cold-weather legumes are a valuable source of premium plant-based protein suitable for human consumption and livestock feed. They play an essential role in crop rotation on arable lands, helping to minimize the requirement for fertilizer usage and acting as effective interim crops [1,2,3,4,5,6]. However, as for any crop, legumes can be affected by a number of diseases, out of which Ascochyta blights are one of the most important groups of necrotic fungal diseases globally present in all legume cultivation areas [7]. Different *Ascochyta* species cause Ascochyta blight diseases in a host-specific manner in many instances: *Ascochyta fabae* Speg., *Ascochyta lentis* Vassiljevsky, *Ascochyta pisi* Lib., *Ascochyta pinodes* (Berk. & Blox.) Jones, *Ascochyta rabiei* (Pass) Labr., and *Ascochyta viciae-villosae* Ondrej are pathogens of faba bean (*Vicia faba* L.), lentil (*Lens culinaris* Medik.), pea (*Pisum sativum* L.), chickpea (*Cicer arietinum* L.), and hairy vetch (*Vicia villosa* Roth), respectively [7,8,9,10,11].

Ascochyta blight management remains problematic, mainly due to the reduced levels of plant resistance available and also because the use of fungicides is uneconomic, forcing the integration of genetic resistance with cultural practices [12,13]. Symptoms generally emerge in the above-ground sections of the plants when exposed to a high percentage of humidity and moderate temperature, resulting in necrotic lesions on both leaves and stems [14,15]. Leaves with multiple lesions tend to fade prematurely, particularly in the lower sections of the plants. On diseased stems, these fungi induce extensive necrotic lesions, which can result in stem breakage and the demise of plant portions situated above the affected area. The infection can also spread through contaminated grains and pods, posing a risk to subsequent crops, as their use can have detrimental effects on the growth of emerging plants. In this study, we focus on the Ascochyta blight of faba bean which is widespread and can cause significant damage by breaking stems, leaf lesions, and seed depreciation. Disease control through crop rotation, clean seed, and chemical treatment is not wholly effective [4] and only moderate levels of genetic resistance are available [16,17], reinforcing the need to understand pathogenicity factors as targets both for resistance breeding and for designing alternative management strategies.

A histological examination revealed that cellular damage and collapse occurred prior to direct fungal contact aimed at breaking down host tissues for nutrient acquisition [18]. To achieve this, necrotrophic fungi can suppress plant defences by releasing harmful substances, primarily enzymes that catalyze the breakdown of structural components and other vital compounds, as well as phytotoxins that induce cell damage and modifications. Nevertheless, the phytotoxic compounds produced by *Ascochyta* associated with legumes are often host specific or exhibit toxicity toward various plants, including their respective hosts. The precise roles of these compounds in the pathogenic process remain unknown [19].

Several metabolites with cytotoxic capacity involved in the pathogenesis process have been found in different *Ascochyta* species. In detail, a range of polyketide-derived secondary metabolites were isolated from the organic extracts of *A. lentis*, *A. pinodes*, and *A. pisi* and chemically characterized by 1D and 2D NMR spectroscopy and mass spectrometry.

Previously, only one metabolite named ascochitine, an *o*-quinone methide, has been identified in *A. fabae* [20]. However, pathogenicity studies have shown that ascochitine is not crucial for causing disease in faba beans, and there is no apparent correlation between the amount of ascochitine and the aggressiveness of *A. fabae* isolates [21]. This finding suggests that other phytotoxic metabolites may be produced by *A. fabae* that play a role in pathogenicity.

To obtain new insight on the interaction between *A. fabae* with its host plants and to obtain new insights into the role of secondary metabolites involved in pathogenicity, we conducted a comprehensive study to isolate and characterize the phytotoxic secondary metabolites produced by *A. fabae* under in vitro conditions. *A. fabae* was grown here in three common culture media to explore their influence on secondary metabolite production. Crude organic extracts from the cultures were subjected to bioassays on the primary host (faba bean) and related legumes of the genera *Vicia*, *Lens*, and *Pisum*. Following this, the organic extracts were purified using chromatographic methods, and spectroscopic techniques, essentially NMR, were employed to dereplicate and characterize the most abundant secondary metabolites fully. Finally, the phytotoxicity of the purified compounds was assessed to gain valuable insights into their roles in fungal pathogenesis.

## 2. Results

*Ascochyta fabae* isolate Af-CO99-01 was grown in vitro in three different substrates (two liquid media, Czapek-Dox and PDB, and one solid rice culture, as detailed in the Section 4) to explore the production of secondary metabolites. After extraction, the phytotoxicity of the three corresponding organic residues on the primary host, faba bean, and other related legumes of economic importance was assayed at different concentrations.

### 2.1. Bioassays of Fungal Organic Extract

**Figure 1:**
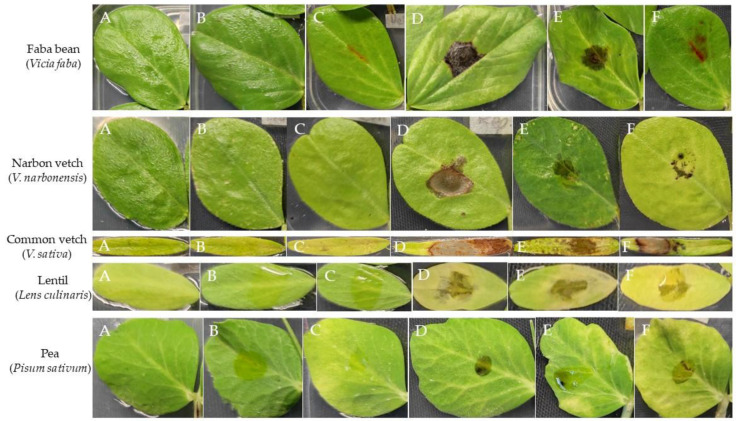
Symptoms developed on detached leaves of several legume hosts as a consequence of the following treatments: (**A**) uninoculated, (**B**) water, (**C**) methanol (MeOH 5%), (**D**) *A. fabae* extract at 2 mg/mL from Czapek-Dox medium, (**E**) *A. fabae* extract at 2 mg/mL from PDB medium and, (**F**) *A. fabae* extract at 2 mg/mL from rice substrate.

The three organic extracts exhibited varying degrees of phytotoxicity, which depended on the fungal growth medium, the applied concentration, and the specific plant species. All three fungal extracts displayed significantly higher phytotoxicity (assessed as foliar damaged area, mm^2^) compared to the controls. The faba bean was the legume crop with the most significant damage, regardless of the culture media employed. Both narbon and common vetches were also significantly damaged, while disease symptoms were generally lower on lentil and pea leaves (Figure 1, Table 1, Supplementary Table S1).

**Figure 2:**
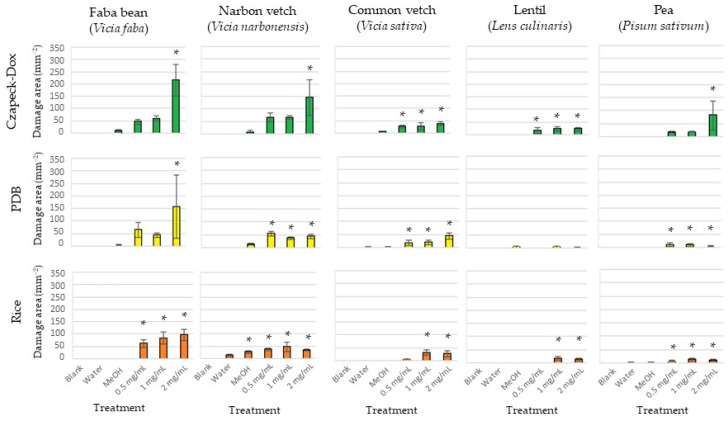
Diseased area (mm^2^) measured on detached leaves of 5 legume crops treated with exudates from the fungus *Ascochyta faba* growth in vitro on 3 different culture media as: Czapek-Dox (green), potato dextrose broth = PDB (yellow) and rice (orange) at concentrations of 0.5, 1, and 2 mg/mL. Negative controls (blank untreated, water and MeOH 5%) were also included. The experiment was repeated four times. Asterisk (*) indicates values significantly different from control MeOH 5%.

Regardless of the host species, the fungal exudate from the Czapek-Dox medium caused higher disease symptoms, followed by exudates from PDB and rice culture (Figure 2, Table 1, Supplementary Table S1). In addition, dose-dependent differences were also observed, especially for the exudate from the Czapek-Dox medium on faba bean, narbon vetch, and pea, as well as for exudate from PDB on faba bean, being the higher dose applied (2 mg/mL) the most phytotoxic. Other treatments did not show a dose-dependent effect.

Given the phytotoxicity exhibited by all three fungal exudates, with the Czapek-Dox extract being the most active among all legume species tested, a comprehensive analysis was conducted to determine the specific metabolite composition displayed by each exudate. The objective of this analysis was to elucidate the distinct metabolic profiles inherent to each exudate. This investigation was undertaken to elucidate potential commonalities or disparities within the exudates, shedding light on the underlying factors contributing to the different phytotoxic effects observed.

### 2.2. Identification of Secondary Metabolites from Culture Filtrates of A. fabae Cultures

Due to the different phytotoxicity displayed by the organic extracts derived from the Czapek-Dox, PDB, and rice cultures observed on both host and no-host legume crops, their purification was carried out using chromatographic techniques, as detailed in the materials and methods section. The predominant metabolites synthesized by the fungus in each culture medium were identified through a comprehensive analysis of NMR spectroscopy and high-resolution LC/MS spectra.

**Figure 3:**
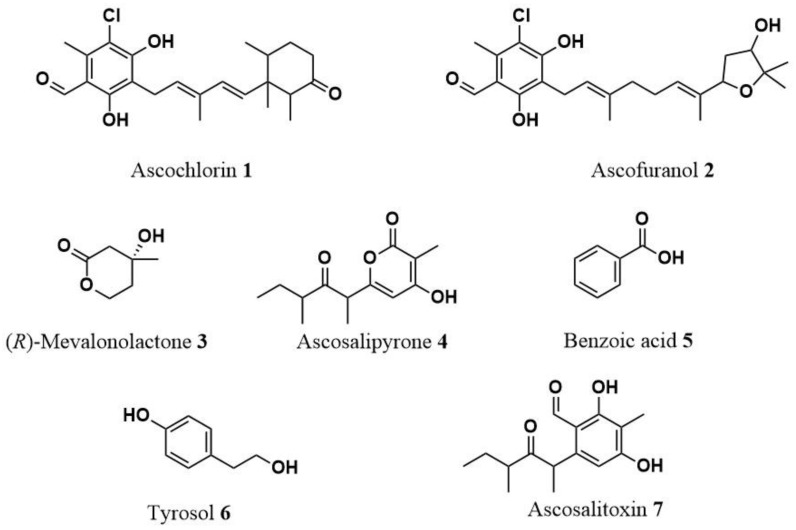
Structure of ascochlorin (**1**), ascofuranol (**2**), (*R*)-mevalonolactone (**3**), ascosalipyrone (**4**), benzoic acid (**5**), tyrosol (**6**), ascosalitoxin (**7**).

From the Czapek-Dox culture, three distinct metabolites were purified: ascochlorin (**1**), ascofuranol (**2**), and (*R*)-mevalonolactone (**3**). Figure 3 illustrates their respective structures. By comparing their spectroscopic properties to the existing literature data, compounds **1**–**3** were successfully identified and dereplicated. Ascochlorin (**1**) was determined to have a molecular formula of C_23_H_29_ClO_4_ based on its HR-ESIMS, revealing two identifiable mass adducts: [M+H]^+^ and [M+Na]^+^. The structural assignment was further confirmed by ^1^H-NMR spectra, which exhibited diagnostic peaks such as the aldehyde proton at δ 10.14, chelated OH at δ 12.72, one aromatic methyl group signal at δ 2.57, and the other four methyl group signals at δ 1.92, 0.83, 0.81, and 0.70. Similarly, ascofuranol (**2**) was found to possess a molecular formula of C_23_H_31_ClO_5_ based on HR-ESIMS, with two identifiable mass adducts: [M+H]^+^ and [M+Na]^+^. The structural assignment was supported by ^1^H-NMR spectra, highlighting diagnostic peaks such as the aldehyde proton at δ 10.14, chelated OH at δ 12.70, one aromatic methyl signal at δ 2.60, geminal dimethyl groups at δ 1.28 and 1.21, and olefinic protons resonating at δ 5.49 and δ 5.15. (*R*)-Mevalonolactone (**3**) had a molecular formula of C_6_H_10_O_3_ as deduced from its HR-ESIMS from the [M+K]^+^ adduct and the dimer [2M+Na]^+^. ^1^H-NMR spectra confirm the structural assignment for the presence of diagnostic peaks: a singlet methyl group at δ 1.28 and the two diastereotopic protons at δ 4.62 and δ 4.41. All the spectra are reported in Supplementary Figures S1–S6.

The PDB culture yielded three unique metabolites: ascosalipyrone (**4**), benzoic acid (**5**), and tyrosol (**6**), that were dereplicated according to their spectroscopic properties reported in the literature (illustrated in Supplementary Figures S7–S12 provide the ^1^H-NMR and ESI/MS spectra). Ascosalipyrone (**4**) was determined to have a molecular formula of C_13_H_18_O_4_ based on its HR-ESIMS, revealing two identifiable mass adducts: [M+H]^+^ and the dimer [2M+Na]^+^. The structural assignment was further confirmed by ^1^H-NMR spectra, which exhibited diagnostic peaks such as the deshielded olefinic proton at δ 5.95, two multiplets at δ 2.69 and δ 1.68, and the four signals of methyl groups at δ 1.94, 1.38, 1.05, and 0.82.

The two simple aromatic compounds benzoic acid (**5**) and tyrosol (**6**) were determined to have molecular formulas C_7_H_6_O_2_ and C_8_H_10_O_2_, respectively. The HR-ESIMS of compound **5** revealed two adducts [M-H_2_O+H]^+^ and [M+H]^+^, while for compound **6**, the adduct [M-H_2_O+H]^+^ and the dimer [2M+H]^+^ were again detected (Supplementary Figures S10 and S12). The structural assignment of benzoic acid (**5**) was further confirmed by ^1^H-NMR spectra, which reveal the typical pattern of monosubstituted benzene with one doublet at δ 8.10 of the two *ortho*-equivalent protons and two triplets at δ 7.62 and δ 7.48. The ^1^H-NMR spectrum of tyrosol (**6**) showed the typical signal pattern of a para-disubstituted benzene with two doublets, each for two equivalent protons, at δ 7.10 and 6.79, and the two triplets of the two methylene of the 2-hydroxy ethyl residue at δ 3.82 and 2.8.

Lastly, the rice culture produced two distinct metabolites: ascosalipyrone (**4**) and ascosalitoxin (**7**) (illustrated in Figure 3). Also, compound **7** was dereplicated by comparing the spectroscopic properties with those reported in the previous study. Supplementary Figures S13 and S14 display its ^1^H-NMR and ESI/MS spectra. Ascosalitoxin (**7**) was determined to have a molecular formula of C_13_H_18_O_4_ based on its HR-ESIMS, revealed by the identifiable mass adduct [M+H]^+^. The structural assignment was further confirmed by ^1^H-NMR spectra, which exhibited diagnostic peaks such as the aldehyde proton at δ 10.23, chelated OH at δ 12.68, one aromatic proton signal at δ 6.23, and the four methyl group signals at δ 2.11, 1.43, 1.08, and 0.72, respectively.

All the dereplicated metabolites have been previously reported as fungal metabolites. Nevertheless, their presence in *A. fabae* in vitro cultures is being reported here for the first time.

### 2.3. Bioassay of Pure Compounds

**Figure 4:**
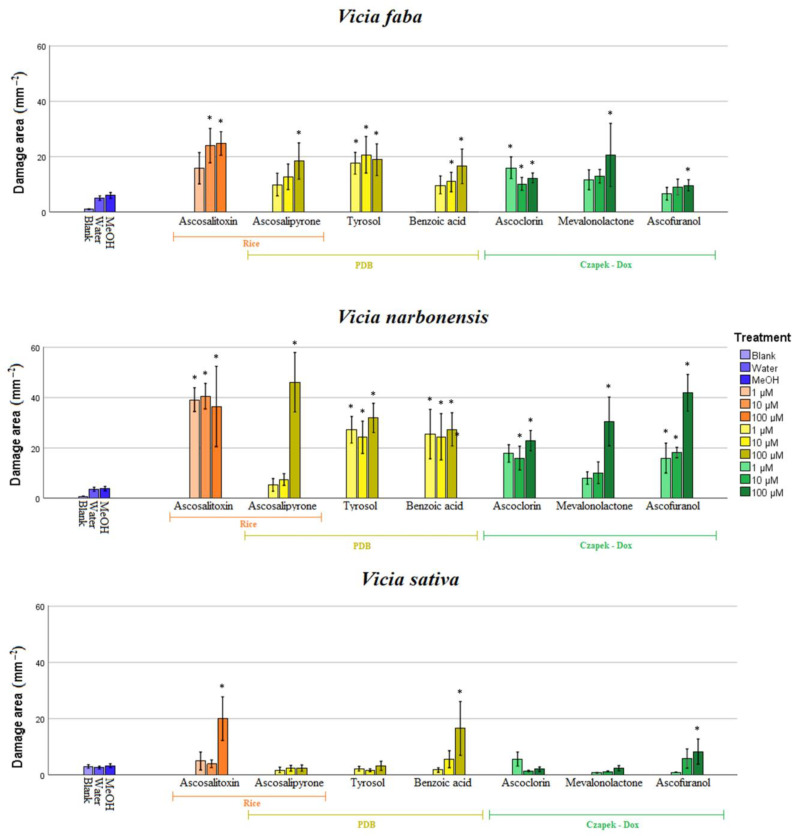
Necrotic area (mm^2^) incited by metabolites **1–7** at different concentrations (1, 10, and 100 µM showed as increased color intensity) on leaves of *Vicia faba*, *V. narbonensis*, *V. sativa* (LSD test, *p* < 0.01). In orange metabolites isolated from rice substrate, in yellow, metabolites isolated from PDB culture; in green, metabolites isolated from Czapek-dox; in blue, negative control: blank uninoculated, water and MeOH 5% controls. Asterisk (*) indicates values significantly different from control MeOH 5%.

The degree of phytotoxicity caused by pure metabolites from *A. fabae* grown on Czapek-Dox, PDB, and rice substrates varied according to the host species and the applied concentration. In general terms, faba bean and narbon vetch were the most susceptible hosts to all the metabolites applied and at any concentrations, followed by common vetch (Figure 4, Supplementary Table S2, Supplementary Figure S15). By contrast, low or no phytotoxicity was induced in both lentil and pea leaves, not significantly different from the negative controls (Supplementary Table S2, Supplementary Figure S15).

Observed by the legume host, species belonging to the genus *Vicia* were the most susceptible (faba bean, narbon vetch, and common vetch), while in pea and lentil hosts, poor or no phytotoxicity was observed. As expected, faba bean was susceptible to all metabolites, at least at the highest concentration tested. In particular, ascosalitoxin (**7**) and benzoic acid (**5**) cause damaged areas of 29.8 and 30.8 mm^2^ and 15 and 16 mm^2^ at applied concentrations of 10 and 100 µM, respectively. Tyrosol (**6**) was phytotoxic at any concentration tested, with damaged areas higher than 17 mm^2^. The other metabolites tested were also phytotoxic, especially at 100 µM. From our results, narbon vetch was the most susceptible legume species tested here, susceptible to all the pure metabolites but with resulting necrotic lesions bigger than those measured in faba bean (Figure 4; Supplementary Table S2; Supplementary Figure S15). Phytotoxicity from ascochlorin (**1**), ascofuranol (**2**), benzoic acid (**5**), tyrosol (**6**), and ascosalitoxin (**7**) was not dose dependent, showing activity at any concentration tested. By contrast, (*R*)-mevalonolactone (**3**) and ascosalipyrone (**4**) showed phytotoxicity only at the higher concentration tested (values higher than 30 and 46 mm^2^, respectively). Common vetch was less affected by the metabolite’s application, showing significant necrotic areas only with ascofuranol (**2**), benzoic acid (**5**), and ascosalitoxin (**7**) at the highest concentration rate.

## 3. Discussion

Among the diseases affecting legumes, Ascochyta blight, incited from the fungal pathogen *Ascochyta fabae*, is one of the most critical necrotic diseases globally present in all legume cultivation areas [17]. Numerous studies suggest that symptoms associated with Ascochyta blight disease seem to be triggered when there is a shift in host physiology, particularly during periods of plant tissue stress [19]. In fact, various chemical and physical factors, whether directly or indirectly, play a role in activating metabolic pathways, which may include the phytotoxic secondary metabolites generated by the fungus. The legume-associated *Ascochyta* spp. produce different metabolites with pathogenesis-determining cytotoxic capacity, many of which display significant toxicity to plants [19,22]. To shed light on the interaction between *A. fabae* and its host plants and to obtain new insights into the role of secondary metabolites involved in pathogenicity, we conducted a comprehensive study to isolate and characterize the most abundant phytotoxic secondary metabolites produced by this pathogen under in vitro conditions. Due to the difference found in the bibliography concerning the culture media described for isolation of phytotoxins produced by *Ascochyta* spp. [19,22,23,24], our study was conducted growing *A. fabae* on three commonly used growth media: PDB, Czapek-Dox, and rice substrate. Despite variations in culture media and substrates employed, the mycelial growth and spore production performed well. The mycelium initially displayed a pale cream color, transitioning into shades of greyish white, dark greenish, and creamy white, aligning with expectations. However, the subsequent investigation into the metabolic profile revealed the significant impact of cultural conditions on the production of secondary metabolites. This outcome is consistent with prior research involving the One Strain Many Compounds Strategy (OSMAC) applied to other fungal and bacteria species [25]. Still, this is the first time that this strategy has been applied to an isolate of *A. fabae*. Exploring diverse cultural conditions is essential for comprehensively exploring the selected microorganism’s chemical space and biosynthetic pathways, effectively simulating *in vivo* conditions. This is particularly relevant in chemical ecology studies aimed at elucidating the role of specialized metabolites in host–pathogen interactions and identifying chemical biomarkers for early disease detection [25,26].

After filtration of the mycelium and extraction of the cultures, the phytotoxicity of each resulting fungal extracts was tested on the host plant and related legumes. The bioassays revealed differences among the extracts, with that obtained from the Czapek-Dox medium being the most active, followed by that from PDB, and finally, from rice substrate. Regarding the host susceptibility, species belonging to the genus *Vicia* spp. were the most susceptible to all the extracts, with faba bean showing the most damage, as expected, since it is the primary host, followed by narbon vetch and common vetch. Lentils and peas, on the other hand, displayed lower susceptibility to the phytotoxic activity of the extracts. These results align with previous findings from cross-inoculation studies with different *Ascochyta* spp. isolates on a panel of legume hosts [11]. The authors found that *A. fabae* principally infected beans (common bean, faba bean, and soybean) and common vetch, while negligible damages were observed in both peas and lentils. These results suggested that the specificity observed might be attributed to bioactive secondary metabolites in the extracts, which could play a role in specific host interactions.

Indeed, when chemical investigation was carried out on the three organic extracts, differences in metabolic composition were appreciated. The prevalent metabolic constituents produced during the in vitro growth by the Ascochyta blight pathogen were isolated by chromatographic methods and dereplicated by extensive spectroscopic studies. As a result, the main compounds identified were ascochlorin, ascofuranol, (*R*)-mevalonolactone, ascosalipyron, benzoic acid, tyrosol, and ascosalitoxin. This was the first time where these metabolites had been purified and dereplicated from an *A. fabae* isolate. Still, their isolation and amount highly depended on the selected cultural condition. In detail, we isolated and dereplicated ascochlorin and ascofuranol together with (*R*)-mevalonolactone in the Czapek-Dox medium. In contrast, ascosalipyron, benzoic acid and tyrosol were isolated and dereplicated in the PDB medium. Finally, when *A. fabae* was grown on rice substrate, the main constituents of the extract were ascosalipyron and ascosalitoxin.

Ascochlorin and ascofuranol are the class natural compounds of meroterpenoids of polyketide–terpene hybrid origin. Ascochlorin was originally isolated from a culture extract of *A. viciae* [27] and later from the entomopathogenic fungus *Verticillium hemipterigenum* [28]. This compound bears a structural resemblance to ubiquinol, an integral component of the respiratory chain for ATP synthesis, exerting inhibitory effects on protozoan alternate oxidase at the ubiquinol-binding site [29]. Notably, ascochlorin has demonstrated diverse biological activities, including antiviral and antibiotic properties, as evident in studies targeting *Candida albicans* [27]. Additionally, it hinders the respiratory chain of ascomycetes yeast *Pichia anomala* by affecting the coenzyme Q [30]. Furthermore, it functions as a non-toxic anticancer agent by inducing G1 cell cycle arrest through p21 induction in a c-Myc-dependent manner rather than p53-dependent [31]. Ascofuranol, a derivative of ascofuranone, was initially isolated from *A. viciae* [32] and was later identified in *A. rabiei* extracts [33]. Ascofuranol exerts its inhibitory action on the alternative oxidase of Trypanosoma by targeting the ubiquinol-binding domain [32]. Ascosalitoxin is a trisubstituted salicylic aldehyde derivative and a biosynthetic precursor of the ascochitine [21]. While this compound was initially isolated from *Ascochyta pisi* [23], it has also been discovered from an endophytic fungus isolated from the medicinal plant *Hintonia latiflora* [34]. Ascosalitoxin has demonstrated cytotoxic activity against human tumor cell lines, manifesting inhibitory effects on the HL-60 cell line [35]. Ascosalipyrone, a polyketide, was first isolated from *A. salicorniae* [36]. It displays potential biological activity as an inhibitor of protein phosphatases [37]. Additionally, it shows antiplasmodial activity against the K1 and NF 54 strains of *Plasmodium falciparum* in conjunction with antimicrobial activity and inhibition of the tyrosine kinase p56lck [36]. In our study, ascosalipyrone was dereplicated from PDB and rice substrate extracts. Notably, the former exhibited higher phytotoxic activity, possibly attributable to the lower production of ascosalipyrone on the rice substrate. In the PDB fungal extracts, two other metabolites were also found: tyrosol and benzoic acid, two simple phenolic metabolites synthesized via the shikimate and phenylpropanoid pathways. They could contribute to infection and phytotoxicity. Nevertheless, it is essential to acknowledge that these metabolites might also fulfill other roles in disease progression or fungal development. Tyrosol is a derivative of phenethyl alcohol. While it has previously been isolated from *A. lentis* and *A. lentis* var. *lathyri* [22], this study marks its first isolation from *A. fabae*. Tyrosol is recognized as a phytotoxic metabolite isolated from both plants and fungi [38]. It frequently appears in cultures of botryosphaeraceous fungi [38] and has been associated with “quorum sensing” in the human pathogenic fungus *Candida albicans* [39]. Tyrosol exhibits antioxidant and anti-inflammatory properties [40] and protects against oxidative stress in renal cells alongside hydroxytyrosol [41]. Furthermore, studies have linked the cytotoxicity of tyrosol and its derivatives with the inhibition of DNA replication initiation [42]. Its potential suitability for stroke therapy in rats has also been explored [43]. On the other hand, benzoic acid has been identified in *Streptomyces lavandulae* [44,45], as well as in *Lactobacillus plantarum*, where it exhibited antimicrobial activity against Gram-negative bacteria such as *Pantoea agglomerans* (*Enterobacter agglomerans*) and *Fusarium avenaceum* (*Gibberella panacea*). In faba beans infected with Fusarium wilt, benzoic acid has been observed to reduce tissue and cell structure resistance, decrease photosynthesis, and increase cell wall degrading enzyme activity [46]. Additionally, it has been shown to inhibit primary root elongation in *Arabidopsis* seedlings, resulting in reduced sizes [47]. (*R*)-Mevalonolactone, a lactone belonging to the δ-valerolactone class, plays a pivotal role as an essential intermediate in the mevalonate biosynthetic pathway. This biosynthetic route is crucial for synthesizing isoprenoids, versatile compounds with diverse functions in various cellular processes [48,49]. Notably, (*R*)-mevalonolactone has been isolated from different pathogenic and non-pathogenic fungi cultivated in vitro, including *Colletotrichum lupini*, *Diaporthaceae* sp. PSU-SP2/4, *Alternaria euphorbicola*, *Pseudallescheria boydii*, and *Phomopsis archeri* [50,51,52,53]. (*R*)-Mevalonolactone was found to enhance chlorophyll content in Leman fronds and inhibit root elongation in cress. Additionally, it led to a considerable reduction in seed germination of *Pelipanche ramosa* [50]. Nevertheless, no phytotoxic effects have been reported for this metabolite.

All these metabolites underwent a phytotoxicity bioassay on the same panel of legume crops of the crude extracts. Notably, prior to this study, there was a lack of information available in the literature regarding the phytotoxic activity of some of the dereplicated metabolites. They all showed different degrees of phytotoxicity regarding legumes and concentration, with the phytotoxic effect more evident in *Vicia* species. In detail, ascochlorin displays intense phytotoxic activity as necrosis on leaves in both faba bean and narbon vetch. Surprisingly, it does not induce significant damage in common vetch despite being first isolated from *A. viciae*. Ascofuranol has a dose-dependent effect in faba beans and peas, while in narbon vetch, necrotic damages are high at all concentrations tested. Ascosalitoxin was the most active metabolite isolated, inducing necrosis in all legume crops tested except lentils. This agrees with previous studies where phytotoxic activity from ascosalitoxin on pea leaves and pods and on tomato seedlings was described [23]. Ascosalitoxin was extracted from rice extract. Although the fungal rice extract had the lowest activity in *Vicia* spp., the pure compound caused the most damage. The difference in activity between the fungal extract and the pure compound may be due to its concentration in the organic extract. For ascosalipyrone this is the first evidence regarding its phytotoxic activity, as no previously available information was described. It was active on faba bean, narbon vetch and pea leaves, but only at the highest concentration tested (100 µM). Similar behavior was observed for (*R*)-mevalonolactone being the most phytotoxin on faba bean and narbon vetch, showing a dose-dependent effect. Tyrosol displayed no dose-dependent effect against faba bean and narbon vetch, causing significant damage even at the lowest concentration of 1 µM. Nevertheless, contrary to previous results [19], lentil is only slightly susceptible, while no significantly diseased leaves were observed in treated vetch or pea. Since tyrosol is a ubiquitous metabolite in plants, this might exceed the plant’s usual levels, leading to necrosis at even lower compound concentrations. Interestingly, benzoic acid is the only compound that exhibits activity in all leguminous plants, but its behavior varies. In narbon vetch, its activity does not depend on concentration. In contrast, in all others, it does, with faba beans showing activity from 10 µM and lentils, peas, and beans only at their highest concentration applied.

The observed variations in bioassay results between crude extracts and pure metabolites may be ascribed to differences in the tested concentrations and production disparities within the various culture media and the potential synergistic or antagonistic effects that may exist among the metabolites generated in each medium. When dealing with complex mixtures of natural products, the dereplication and identification of the specific components responsible for their activities and comprehending the mechanisms involved in their interactions remains a tricky challenge. Such mechanisms can be multifaceted and vary depending on the cultivation methods, preparation, and processing of these compounds, as observed in previous research [54,55]. Modern analytical chemistry techniques, chemoinformatic tools, and metabolomics play a pivotal role in the dereplication of intricate organic extracts by identifying and cataloguing known metabolites and promoting the search for novel compounds. Additionally, it offers a powerful tool for quantifying minor components within these extracts, aiding in accurately assessing their abundance [56,57,58,59,60]. Furthermore, it is essential to highlight that the low amount and the high number of chiral carbons in ascochlorin ascofuranol, ascosalipyrone, and ascosalitoxin did not allow their complete stereochemical characterization using the spectroscopic method. Existing information on the stereochemistry of these metabolites is limited [23,61]. Future research should prioritize the assignment of chiral carbon configurations to fully elucidate their role in the Ascochyta–legume interaction. The absolute assignment of fungal secondary metabolite configurations through advanced spectroscopic methods, including NMR and optical techniques, is indispensable for comprehensively understanding their biological relevance [62,63,64]. Accurate molecular structure determination provides insights into the compound’s bioactivity and potential ecological roles.

Finally, it is essential to highlight that high-molecular-weight phytotoxins were not studied under the conditions of this research. In previous studies involving other fungal species, hydrophilic high-molecular-weight metabolites, such as polysaccharide peptides with phototoxic properties that could have a role as elicitors, have been detected and studied in vitro [65,66,67].

In conclusion, our research has provided insights into the phytotoxic secondary metabolites produced by *A. fabae* under varying in vitro conditions, unveiling a rich diversity of compounds with differential phytotoxic effects. Notably, prior to this study, only ascochitine was known to be produced by *A. fabae*. We have dereplicated a panel of seven metabolites belonging to different classes of natural compounds that could be used for targeted in-depth investigations. Nevertheless, the observed variations in bioassay results between crude extracts and pure metabolites underline the intricate nature of these interactions, influenced by synergistic or antagonistic effects among the metabolites and further studies using more sensitive techniques are also needed to identify the minor constituents of the fungal exudate. These findings pave the way for further research to elucidate these seven metabolites’ underlying mechanisms and ecological implications in legume–plant interactions. Understanding these complex relationships is essential for advancing our knowledge of host–pathogen interactions and developing more specific strategies for Ascochyta blight management and early detection.

## 4. Materials and Methods

### 4.1. Fungal Strain, Culture Medium and Growth Conditions

A previously well-characterized monoconidial strain of *Ascochyta fabae* (Af-CO99-01) [68], isolated from a diseased faba bean (*Vicia faba*) crop and belonging to the fungal collection maintained at the Institute for Sustainable Agriculture (IAS-CSIC, Córdoba, Spain), was selected for these experiments. The pathogen was preserved in sterile cellulose filter papers. Before the experiment, inoculum was prepared by multiplying spores of the isolate on potato dextrose agar (PDA) (Sigma Aldrich, Saint-Quentin Fallavier, France) medium under controlled conditions as previously described [11]. Then, the isolate was differentially growth in two artificial media and one solid substrate as follows: (i) 10 flasks containing 1 L of Czapek-Dox medium [69]. Each flask was inoculated with a 1-week-old mycelial plate of the isolate on PDA. The cultures were incubated at 24 °C (stirring conditions, 150 rpm), in absence of light for 21 days. The fungal mycelium was then removed by filtration through four layers of filter paper, centrifuged and kept at −20 °C until the next analysis; (ii) 10 flasks containing 1 L of potato dextrose broth (PDB) (BD Difco^®^, Crystal Lake, NJ, USA) medium. Each flask was inoculated with a 1-week-old mycelial plate of the isolate on PDA and kept at similar conditions to those mentioned in point (i); (iii) 1 L flask containing 400 g of common rice. Water was added to the flask (45%, vol/vol) and allowed 24 h to be absorbed. Then, the material was sterilized at 121 °C for 30 min. Inoculation was carried out with a 1-week-old mycelial plate of the isolate on PDA. The culture was then incubated in conditions described by Reveglia et al. [70].

### 4.2. General Experimental Procedure for Chemical Analysis

Analytical and preparative TLCs were carried out on silica gel (Merck, Kieselgel 60, F254, 0.25, and 0.5 mm) and reverse phase (Merck, Kieselgel 60 RP-18, F254, 0.20 mm) plates. The spots were visualized by exposure to UV radiation (254 and/or 312 nm) or by spraying first with 10% H_2_SO_4_ in MeOH, and then with 5% phosphomolybdic acid in EtOH, followed by heating at 110 °C for 10 min on a hot plate. Column chromatography was performed using silica gel (Merck, Kieselgel 60, 0.063–0.200 mm). Solvents *n*-hexane MeOH, *i*-PrOH, CHCl_3_, and CH_2_Cl_2_ were purchased from Panreac AppliChem (Barcelona, Spain). Unless otherwise noted, optical rotation was measured in MeOH on a Jasco (Tokyo, Japan) polarimeter, whereas the CD spectrum was recorded on a JASCO J-815 CD in MeOH. ^1^H and ^13^C NMR and 2D NMR spectra were recorded at 400 or 500, and 100 or 125 MHz in CDCl_3_ on Bruker and Varian instruments. The same solvent was used as an internal standard. HR-ESIMS analyses were performed using the LC/MS TOF system (AGILENT 6230B, HPLC 1260 Infinity) column Phenomenex LUNA (C18 (2) 5 µm 150 × 4.6 mm). ^1^H-NMR and ESI/MS (+) spectra of the identified compounds are reported in the Supplementary Figures S1–S14.

### 4.3. Extraction and Purification of Secondary Metabolites Produced in Czapek-Dox Culture

The culture filtrates (10 L) of *A. fabae* were lyophilized and dissolved in 1/10 distilled water of the original volume. The solution was exhaustively extracted with EtOAc (3 × 300 mL). The organic extracts were combined, dried (Na_2_SO_4_), and evaporated under reduced pressure. The corresponding residue (439 mg) was purified by silica gel column, eluted with CHCl_3_-*i*-PrOH (95:5), yielding six homogeneous fraction groups. The residue of the first fraction (80 mg) was purified by TLC, eluted with CHCl_3_ affording two white amorphous solids identified as ascochlorin (**1**, 1.7 mg) and ascofuranol (**2**, 2 mg). The residue of the fourth fraction (80 mg) was purified by TLC, eluted with CHCl_3_-*i*-PrOH (97:3), affording a white amorphous solid identified as (*R*)-mevalonolactone (**3**, 1.9 mg).

Ascochlorin (**1**): ^1^H-NMR and ESI/MS (+) data agree with those previously reported [27,71].

Ascofuranol (**2**): ^1^H-NMR and ESI/MS (+) data agree with those previously reported [32].

(*R*)-Mevalonolactone (**3**): ^1^H-NMR and ESI/MS (+) data agree with those previously reported [50].

### 4.4. Extraction and Purification of Secondary Metabolites Produced in PDB Culture

The culture filtrates (10 L) of *A. fabae* were lyophilized and dissolved in 1/10 distilled water of original volume. The solution was exhaustively extracted with EtOAc (3 × 300 mL). The organic extracts were combined, dried (Na_2_SO_4_), and evaporated under reduced pressure. The corresponding residue (441 mg) was purified by silica gel column, eluted with CHCl_3_-*i*-PrOH (95:5), yielding seven homogeneous fraction groups. The residue of the second fraction (34 mg) was purified by TLC, eluted with CHCl_3_-*i*-PrOH (97:3), affording two white amorphous solids identified as ascosalipyrone (**4**, 10 mg), and as benzoic acid (**5**, 8 mg). The residue of the third fraction (60 mg) was purified by TLC and eluted with CHCl_3_-*i*-PrOH (95:5), affording a white amorphous solid identified as tyrosol (**6**, 1.6 mg).

Ascosalipyrone (**4**): ^1^H-NMR and ESI/MS (+) data agree with those previously reported [36].

Benzoic acid (**5**): ^1^H-NMR and ESI/MS (+) data agree with those previously reported. [44].

Tyrosol (**6**): ^1^H-NMR and ESI/MS (+) data agree with those previously reported [72].

### 4.5. Extraction and Purification of Secondary Metabolites Produced in Rice Culture

The solid culture of *A. fabae* (440 g) was subjected to air-drying at 27 °C for a minimum of two weeks before the extraction process. Subsequently, the dried material was finely ground using a laboratory mill and then subjected to extraction with a mixture of 500 mL of MeOH–H_2_O (1% NaCl) in a 1:1 ratio. Afterward, the mixture underwent centrifugation at 10,000 rpm for 1 h. The resulting pellet was subjected to a second round of extraction using the same solvent mixture under identical conditions. The two supernatants obtained were combined, treated with *n*-hexane (2 × 500 mL) for defatting, and further extracted with CH_2_Cl_2_ (3 × 500 mL). The organic extracts in CH_2_Cl_2_ were pooled, desiccated using Na_2_SO_4_, and subsequently concentrated under reduced pressure to produce a brown solid residue weighing 226 mg. The corresponding residue was purified by silica gel column, eluted with CHCl_3_-*i*-PrOH (95:5), yielding six homogeneous fraction groups. The residue of the second fraction (4.7 mg) was purified by TLC eluted with CHCl_3_-*i*-PrOH (97:3). This afforded a white amorphous solid identified as ascosalitoxin (**7**, 0.8 mg). The residue of fraction six (18 mg) was purified by TLC, eluted with CHCl_3_-*i*-PrOH (95:5), yielding another amorphous solid identified as ascosalipyrone (**4**, 1 mg).

Ascosalitoxin (**7**): ^1^H-NMR and ESI/MS (+) data agree with those previously reported [23,34].

### 4.6. Bioassays

The phytotoxic effects of all the *A. fabae* organic extracts and those of pure compounds **1**–**7** were evaluated using a detached leaf method [73]. Several legume crops (listed in Table 2) were selected and grown in chamber as follows: seeds were sown in pots (6 × 6 × 10 cm) filled with a potting mixture (sand/peat, 1:3 vol/vol), then were grown in a growth chamber at 20 ± 2 °C and 65% relative humidity under a photoperiod with 14 h light/10 h dark at a light intensity of 200 μmol m^−2^ s^−1^ photon flux density supplied by high-output white fluorescent tubes until the fifth leaf stage was achieved [70]. Leaves from each legume specie were excised and placed, adaxial side up, on 4% technical agar in Petri dishes. The three organic fungal extracts were dissolved in MeOH (5%) and then added to the assay concentration with distilled water of 0.5, 1, and 2 mg/mL. Similarly, bioassays performed with pure compounds **1**–**7** and were arranged in Petri dishes as described above and assayed at 1, 10, and 100 µM concentrations. For each legume specie, fungal extract, compound, and concentration assayed, cut leaves were arranged in a randomized design with three replicates per treatment, each replicate having four leaves.

### 4.7. Data Analysis

A completely randomized design was used in all detached leaves essays. The presence of symptoms through the appearance of dark spots or discoloration of the plant tissue was monitored, introducing a method of image acquisition by an android smartphone. The smartphone was equipped with CMOS image sensor and SMD LED background light illumination to provide a constant brightness for all the images captured and reduce the effect of ambient lighting condition. Samsung galaxy J2 smartphone (Samsung Engineering Co., Ltd., Seoul, Republic of Korea) was used to acquire 2 images per detached leaves and per plate to be analyzed. All the images collected were in an RGB color space. The damage area (mm^2^) was measured on the smartphone-captured images, with the help of ImageJ (1.46 r) program (free license). The significance of the differences in leaf damage between plant species, treatments, and concentrations was estimated by one-way analysis of variance (ANOVA). All statistical analyses were performed using the Statistix 9.0 package (Analytical Software, Tallahase, FL, USA). Significance of differences between means was determined by calculating least significant difference (LSD).

One-dimensional and two-dimensional NRM data were analyzed and interpreted by MNova software v. 14 (MestreLab Research S.L, Santiago de Compostela, Spain).

## Figures and Tables

**Table 1:** Differences by host specie on disease severity (necrotic area, mm^2^) caused by *Ascochyta fabae* fungal exudates from different culture media (Czapek-Dox, PDB, and rice). Values are the general average of all concentrations tested. Negative controls (blank untreated, water and MeOH 5%) were also included. The experiment was repeated four times.

	Host Plant									
Treatment	Faba Bean		Narbon Vetch		Common Vetch		Lentil		Pea	
Blank	0 ± 0	d	0 ± 0	c	0 ± 0	c	0 ± 0	c	0 ± 0	c
Water	0 ± 0	d	1.8 ± 1.6	c	0.2 ± 0.1	c	0 ± 0	c	0.3 ± 0.2	c
MeOH	3.4 ± 1.5	c	7.3 ± 3.3	bc	0.2 ± 0.05	c	0.5 ± 0.3	c	0.4 ± 0.2	c
Czapek-Dox	110.6 ± 3.9	a	96.4 ± 8.8	a	27.9 ± 5.5	ab	19.9 ± 5.2	a	29.2 ± 3.2	a
PDB	95.6 ± 5.4	b	45.2 ± 4.7	b	30.2 ± 7.3	a	1.0 ± 0.6	c	7.5 ± 2.6	b
Rice	81.6 ± 2.6	b	38.9 ± 5.1	b	17.1 ± 5.1	b	9.1 ± 3.4	b	9.9 ± 2.1	b

Values, per column and treatment, followed by different letters differ significantly at *p* < 0.01.

**Table 2:** Legume species and genotypes grow under controlled conditions and are used in detached leaves assays.

Legume	Plant Specie	Genotype
Faba bean	*Vicia faba*	Baraca
Narbon vetch	*V. narbonensis*	VN01
Common vetch	*V. sativa*	Buzza
Lentil	*Lens culinaris*	Pardina
Pea	*Pisum sativum*	Messire

## Data Availability

All the data that arose from this research are included in the manuscript and in the supplementary material.

## Supplementary Materials

The following supporting information can be downloaded at: https://www.mdpi.com/article/10.3390/toxins15120693/s1. Supplementary Figure S1. ^1^H-NMR spectrum of ascochlorin (**1**) (CDCl_3_, 400 MHz); Supplementary Figure S2. ESI/MS (+) spectrum of ascochlorin (**1**); Supplementary Figure S3. ^1^H-NMR spectrum of ascofuranol (**2**) (CDCl_3_, 400 MHz); Supplementary Figure S4. ESI/MS (+) spectrum of ascofuranol (**2**); Supplementary Figure S5. ^1^H-NMR spectrum of (*R*)-mevalonolactone (**3**) (CDCl_3_, 400 MHz); Supplementary Figure S6. ESI/MS (+) spectrum of (*R*)-mevalonolactone (**3**); Supplementary Figure S7. ^1^H-NMR spectrum of ascosalipyrone (**4**) (CDCl_3_, 400 MHz); Supplementary Figure S8. ESI/MS (+) spectrum of ascosalipyrone (**4**); Supplementary Figure S9. ^1^H-NMR spectrum of benzoic acid (**5**) (CDCl_3_, 400 MHz); Supplementary Figure S10. ESI/MS (+) spectrum of benzoic acid (**5**); Supplementary Figure S11. ^1^H-NMR spectrum of tyrosol (**6**) (CDCl_3_, 400 MHz); Supplementary Figure S12. ESI/MS (+) spectrum of tyrosol (**6**); Supplementary Figure S13. ^1^H-NMR spectrum of ascosalitoxin (**7**) (CDCl_3_, 400 MHz); Supplementary Figure S14. ESI/MS (+) spectrum of ascosalitoxin (**7**); Supplementary Figure S15. Images of the symptoms of each of the compounds. Supplementary Table S1. Diseased area (mm^2^) measured on detached leaves of several legume crops with exudates from the fungus *A. fabae* growth in vitro on 3 different culture media (Czapek-Dox = CD, potato dextrose broth = PDB, and rice) at concentrations of 0.5, 1, and 2 mg/mL. Negative (blank untreated, water and MeOH 5%) controls were also included. The experiment was repeated four times. Supplementary Table S2. Diseased area (mm^2^) measured in leaves detached from various legume crops with metabolites produced by the exudate of the *A. fabae* fungus from the three-growth media at concentrations of 1, 10, and 100 µM. Negative controls (untreated blank, water, and MeOH 5%) were also included. *p*-value compared with value from MeOH 5% control.
