# Supplementary material for: Comparative Analysis of Secondary Metabolites Produced by Ascochyta fabae under In Vitro Conditions and Their Phytotoxicity on the Primary Host, Vicia faba, and Related Legume Crops
Source: Toxins (Basel). 2023 Dec 9;15(12):693. doi: 10.3390/toxins15120693 (PMC10747461; doi:10.3390/toxins15120693)
Supplement: Supplementary file 1 [file toxins-15-00693-s001.zip › toxins-2727251-supplementary.pdf]

Supplementary Materials: Comparative analysis of secondary metabolites produced by *Ascochyta fabae* under *in vitro* conditions and their phytotoxicity on the primary host, *Vicia faba*, and related legume crops

Eleonora Barilli, Pierluigi Reveglia, Francisco Jesús Agudo-Jurado, Vanessa Cañete García, Alessio Cimmino, Antonio Evidente and Diego Rubiales

Supporting Information List

Supplementary Figure 1. ^{1}H NMR spectrum of ascochlorin (**1**) ($CDCl_3$, 400 MHz).

Supplementary Figure 2. ESI/MS (+) spectrum of ascochlorin (**1**).

Supplementary Figure 3. ^{1}H NMR spectrum of ascofuranol (**2**) ($CDCl_3$, 400 MHz).

Supplementary Figure 4. ESI/MS (+) spectrum of ascofuranol (**2**).

Supplementary Figure 5. ^{1}H NMR spectrum of (*R*)-mevalonolactone (**3**) ($CDCl_3$, 400 MHz).

Supplementary Figure 6. ESI/MS (+) spectrum of (*R*)-mevalonolactone (**3**).

Supplementary Figure 7. ^{1}H NMR spectrum of ascosalipyrone (**4**) ($CDCl_3$, 400 MHz).

Supplementary Figure 8. ESI/MS (+) spectrum of ascosalipyrone (**4**).

Supplementary Figure 9. ^{1}H NMR spectrum of benzoic acid (**5**) ($CDCl_3$, 400 MHz).

Supplementary Figure 10. ESI/MS (+) spectrum of benzoic acid (**5**).

Supplementary Figure 11. ^{1}H NMR spectrum of tyrosol (**6**) ($CDCl_3$, 400 MHz).

Supplementary Figure 12. ESI/MS (+) spectrum of tyrosol (**6**).

Supplementary Figure 13. ^{1}H NMR spectrum of ascosalitoxin (**7**) ($CDCl_3$, 400 MHz).

Supplementary Figure 14. ESI/MS (+) spectrum of ascosalitoxin (**7**).

Supplementary Figure 15. Symptoms developed on detached leaves of several legume hosts treated as follow: (a) uninoculated, (b) water, (c) methanol (MeOH 5%), (d) ascoclorin, (e) ascofuranol, (f) (*R*)-mevalonolactone, (g) ascosalipyrone, (h) benzoic acid, (i) tyrosol and (j) ascosalitoxin.

Supplementary Table 1. Diseased area (mm^2) measured on detached leaves of several legume crops with exudates from the fungus *Ascochyta fabae* growth *in vitro* on 3 different culture media (Czapek-Dox = CD, Potato Dextrose Broth = PDB and rice) at concentrations of 0.5, 1 and 2 mg/mL. Negative (blank untreated, water and MeOH 5%) controls were also included. The experiment was repeated four times.

Supplementary Table 2. Diseased area (mm^2) measured in leaves detached from various legume crops with metabolites produced by the exudate of the *Ascochyta fabae* fungus from the three-growth media at concentrations of 1, 10 and 100μM. Negative controls (untreated blank, water and MeOH 5%) were also included. *P*-value compared with value from MeOH 5% control.

Supplementary Figure 1. ^1H NMR spectrum of ascochlorin (**1**) ($CDCl_3$, 400 MHz).

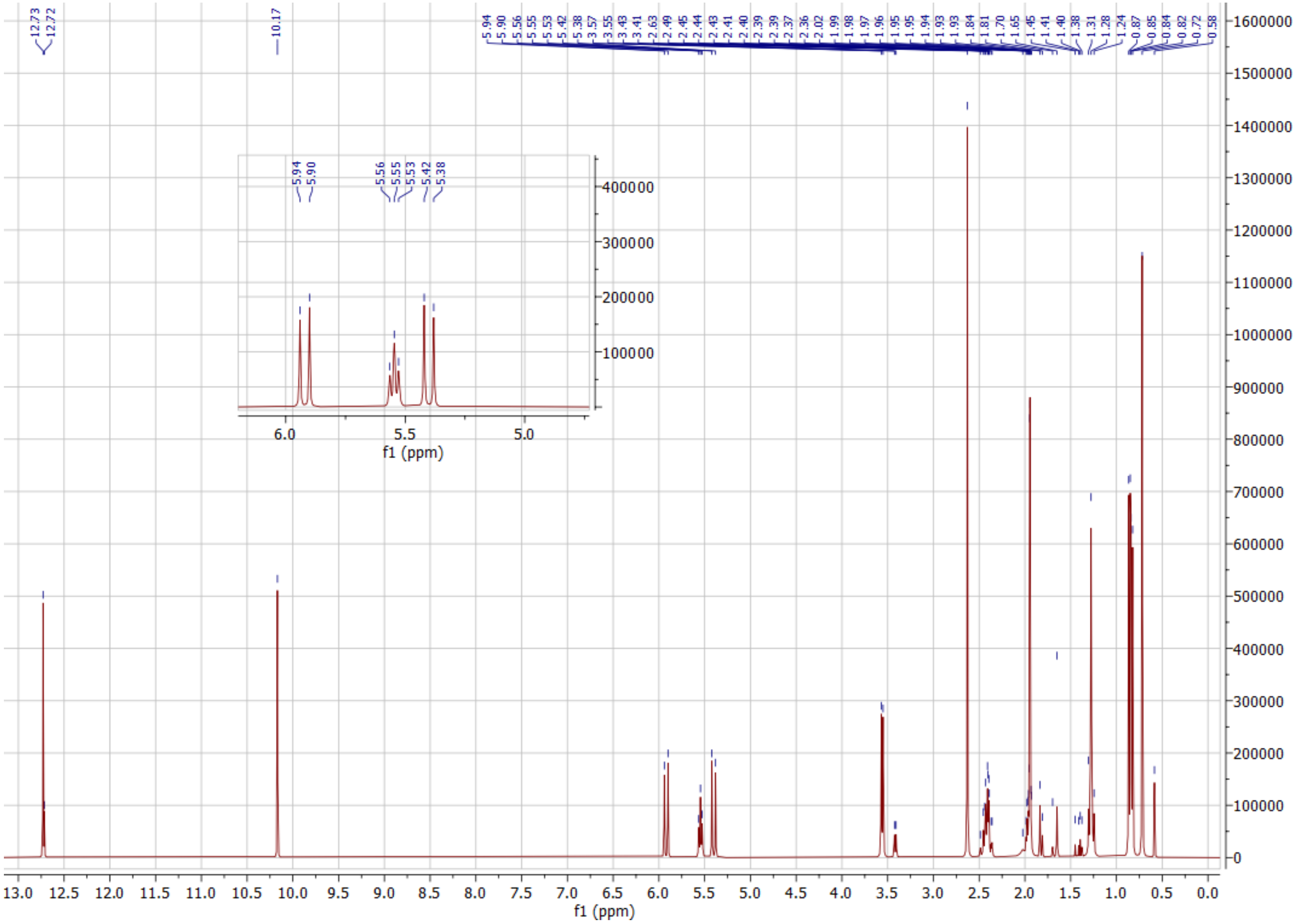

Supplementary Figure 2. ESI/MS (+) spectrum of ascochlorin (**1**).

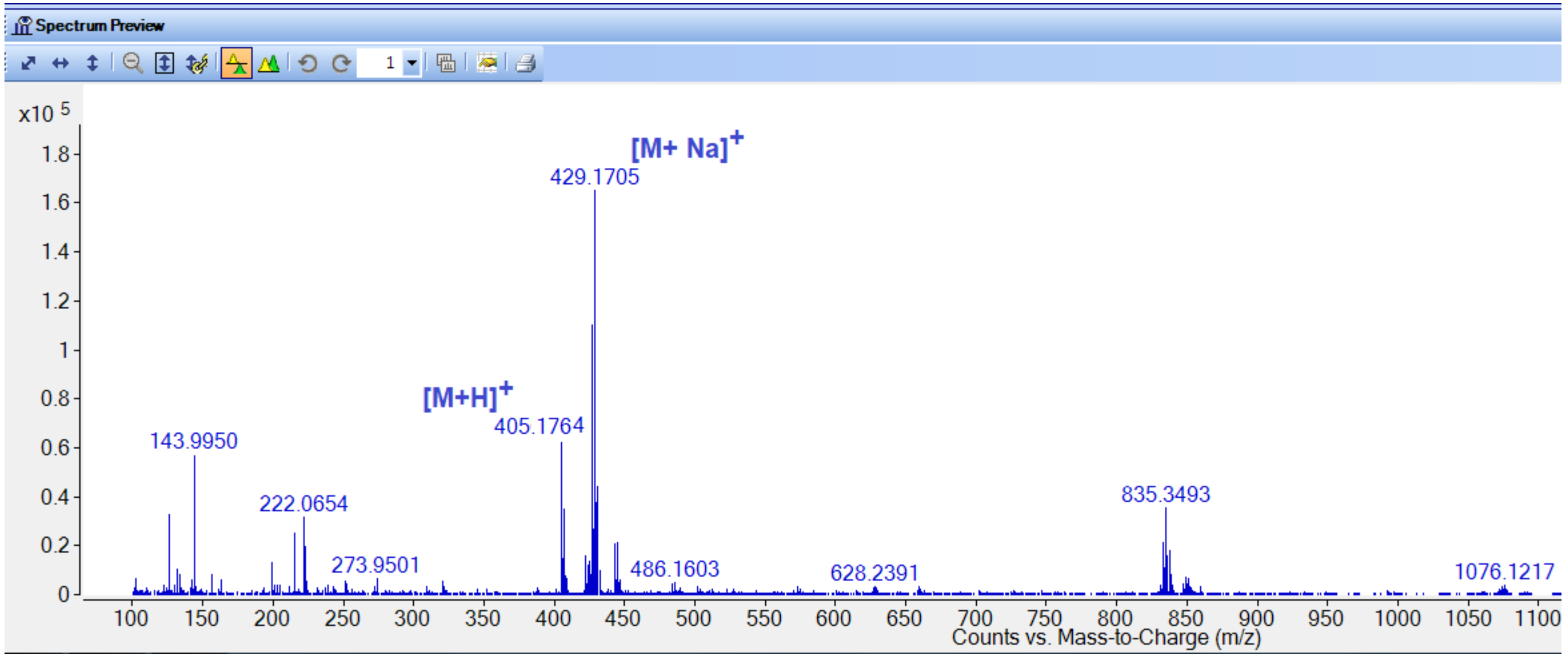

Supplementary Figure 3. ^{1}H NMR spectrum of ascofuranol (**2**) ($CDCl_3$, 400 MHz).

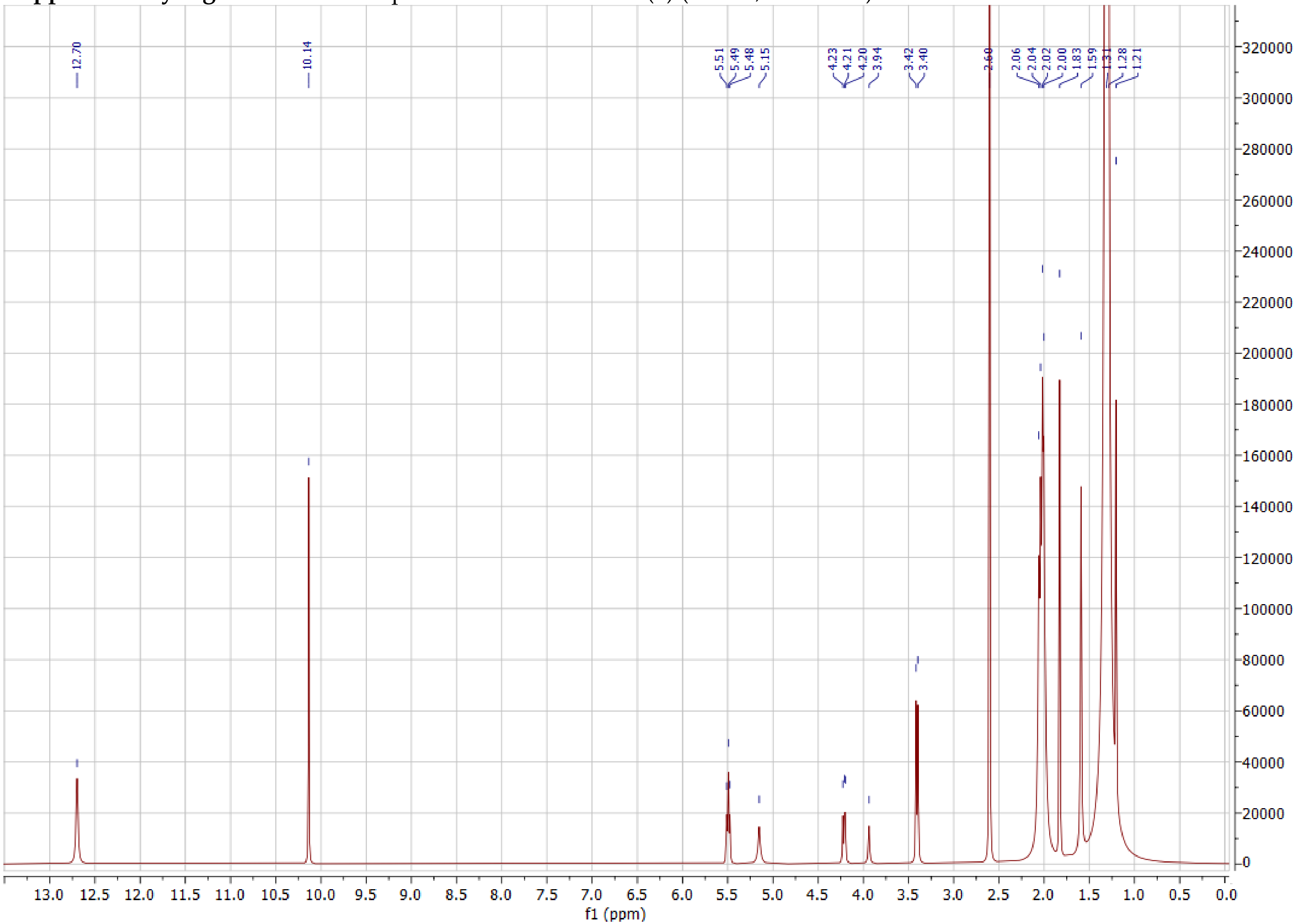

Supplementary Figure 4. ESI/MS (+) spectrum of ascofuranol (**2**).

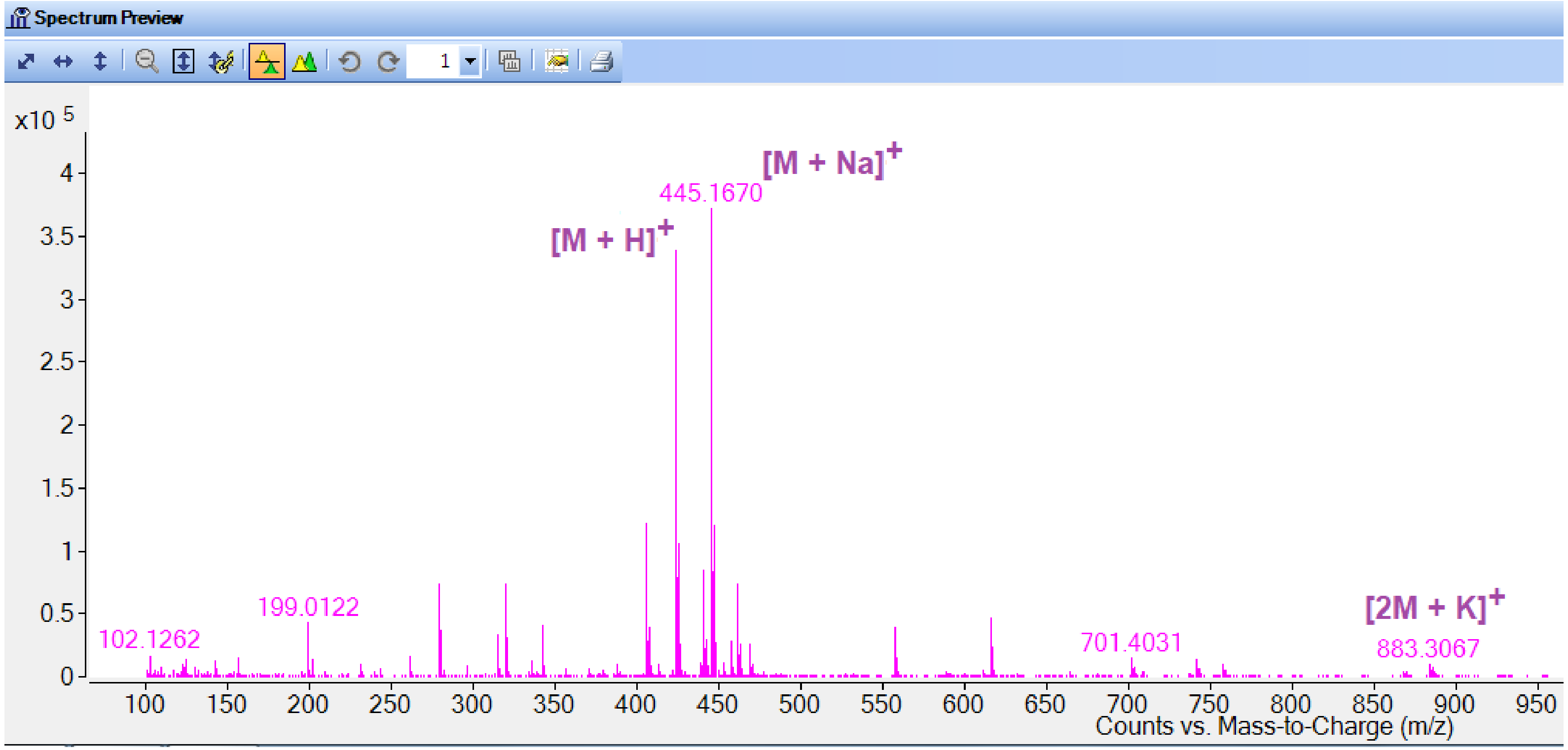

Supplementary Figure 5. ^{1}H NMR spectrum of (*R*)-mevalonolactone (**3**) ($CDCl_3$, 400 MHz).

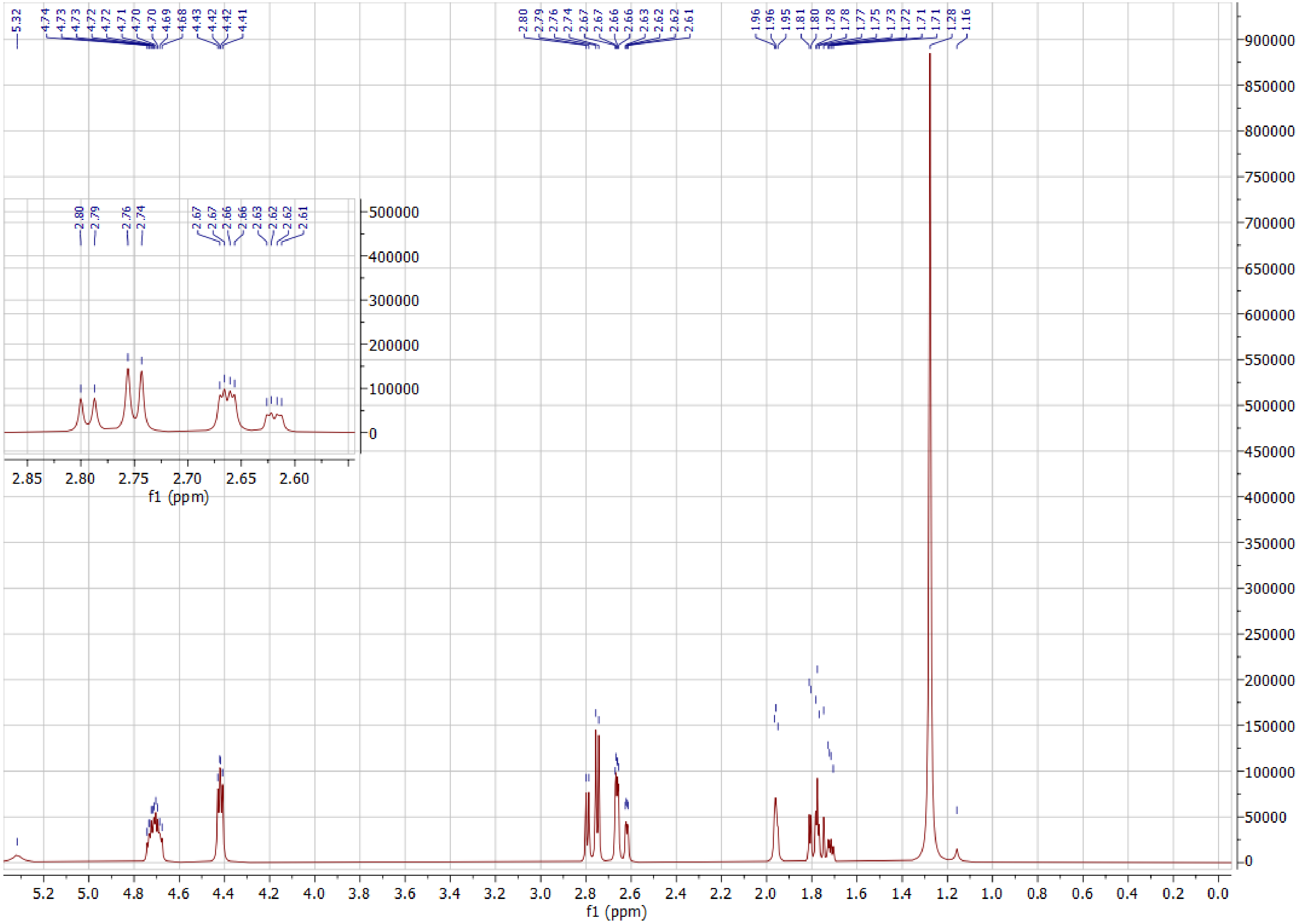

Supplementary Figure 6. ESI/MS (+) spectrum of (*R*)-mevalonolactone (**3**).

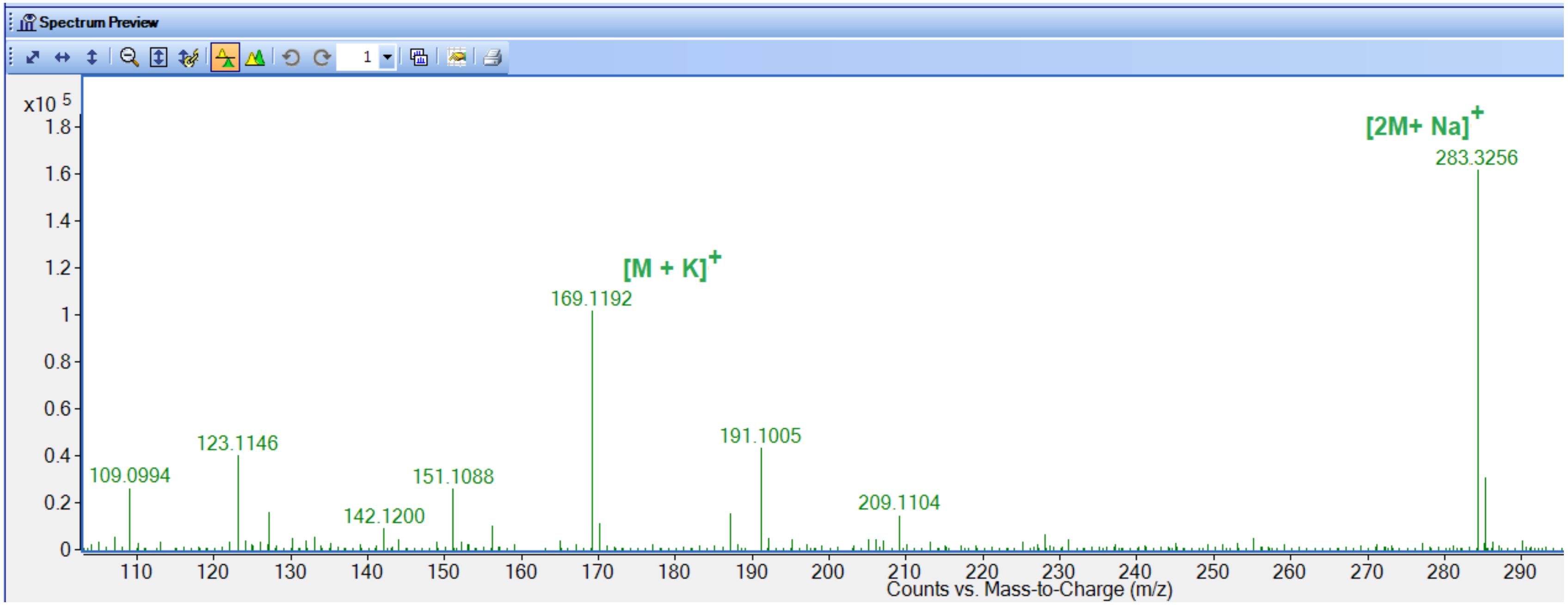

Supplementary Figure 7. ^{1}H NMR spectrum of ascosalipyrone (**4**) ($CDCl_3$, 400 MHz).

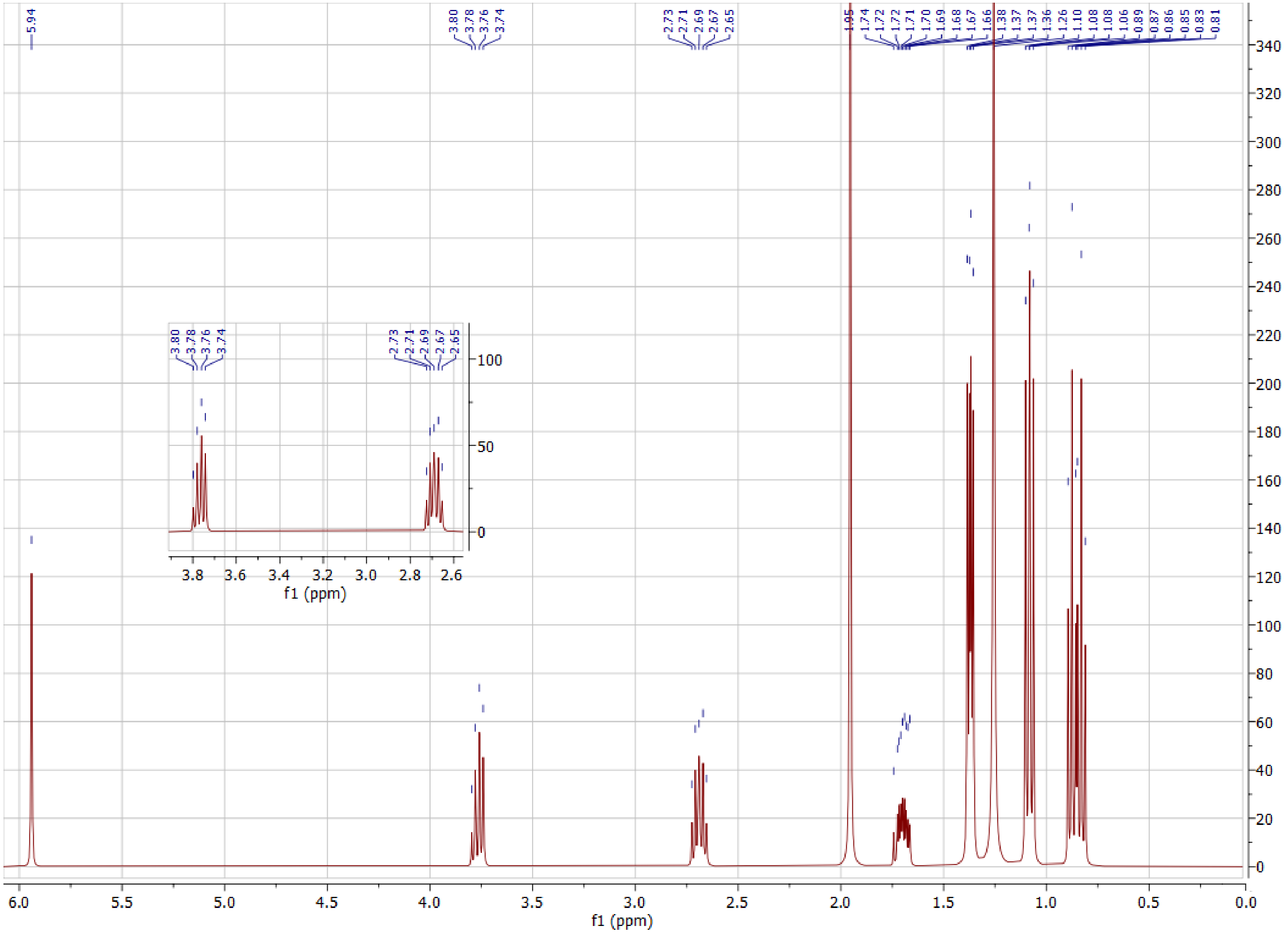

Supplementary Figure 8. ESI/MS (+) spectrum of ascosalipyrone (**4**).

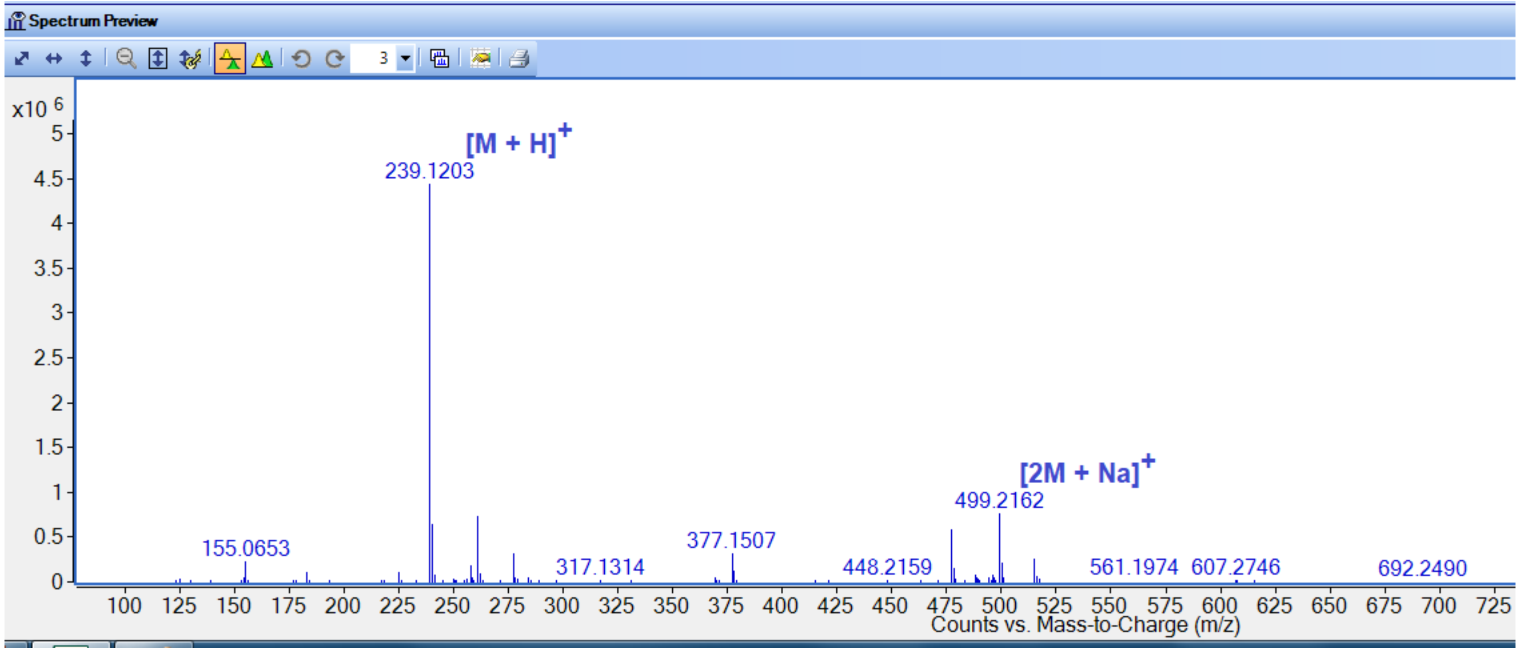

Supplementary Figure 9. ^{1}H NMR spectrum of benzoic acid (**5**) ($CDCl_3$, 400 MHz).

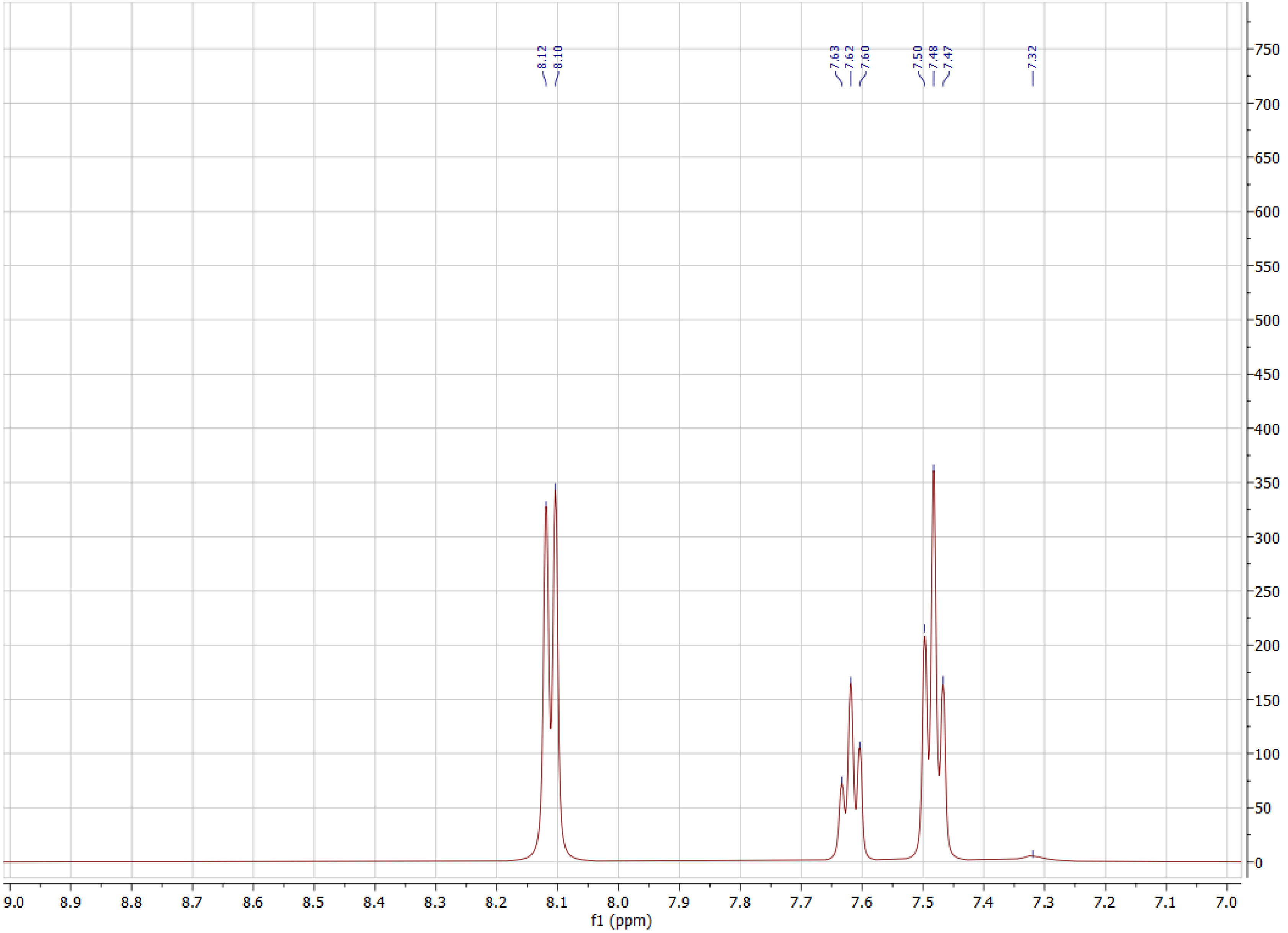

Supplementary Figure 10. ESI/MS (+) spectrum of benzoic acid (**5**).

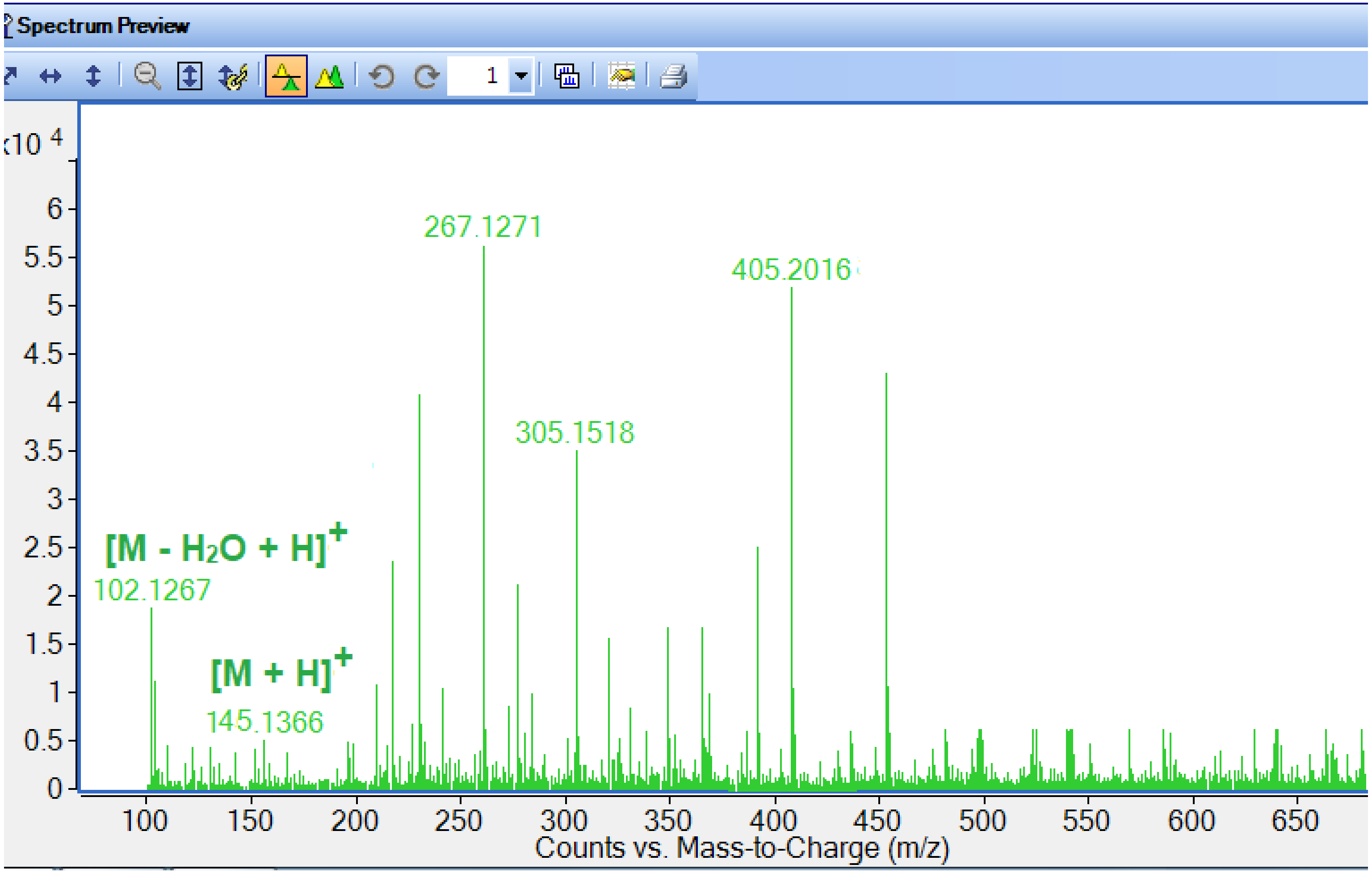

Supplementary Figure 11. ^{1}H NMR spectrum of tyrosol (**6**) ($CDCl_3$, 400 MHz).

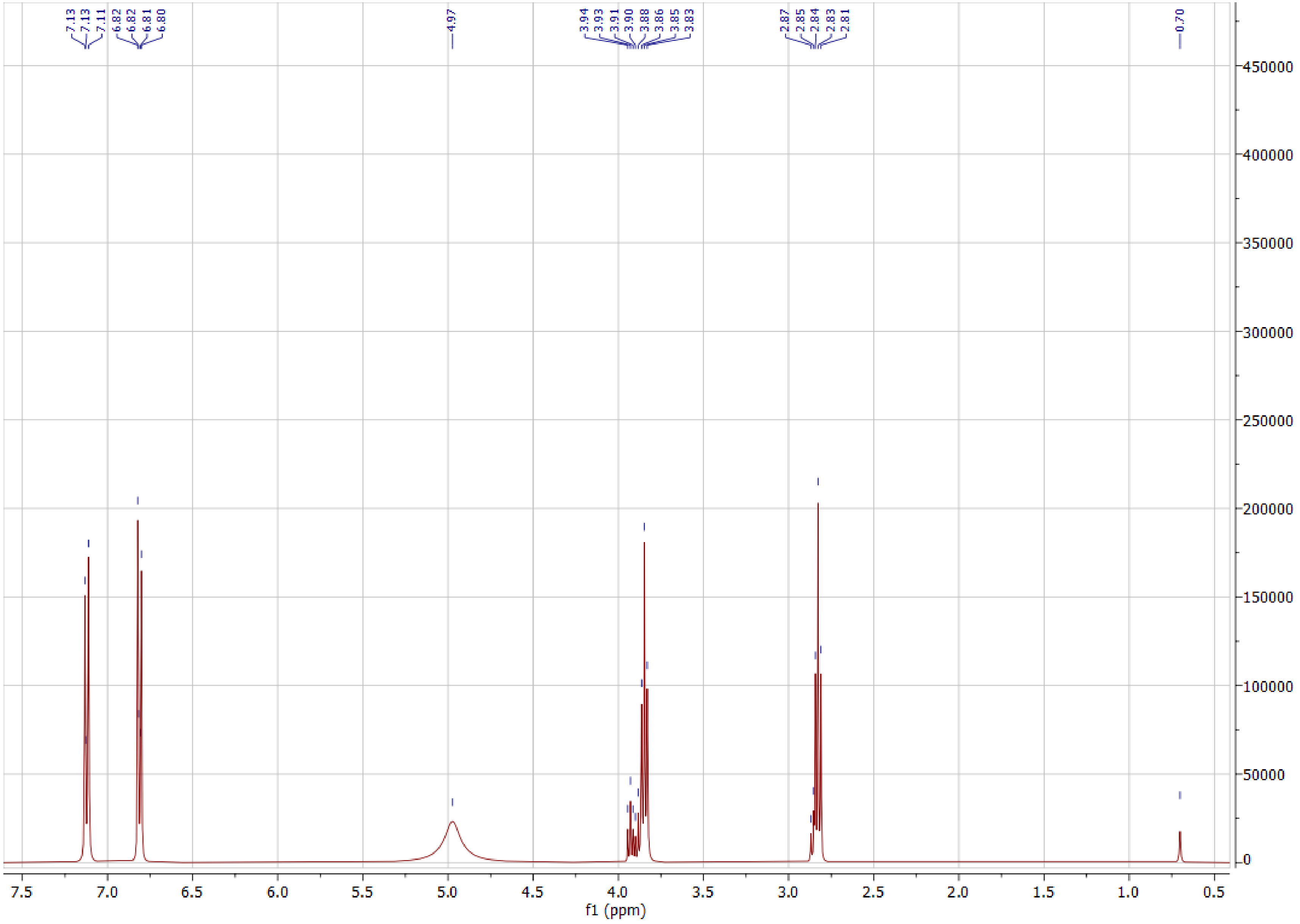

Supplementary Figure 12. ESI/MS (+) spectrum of tyrosol (**6**).

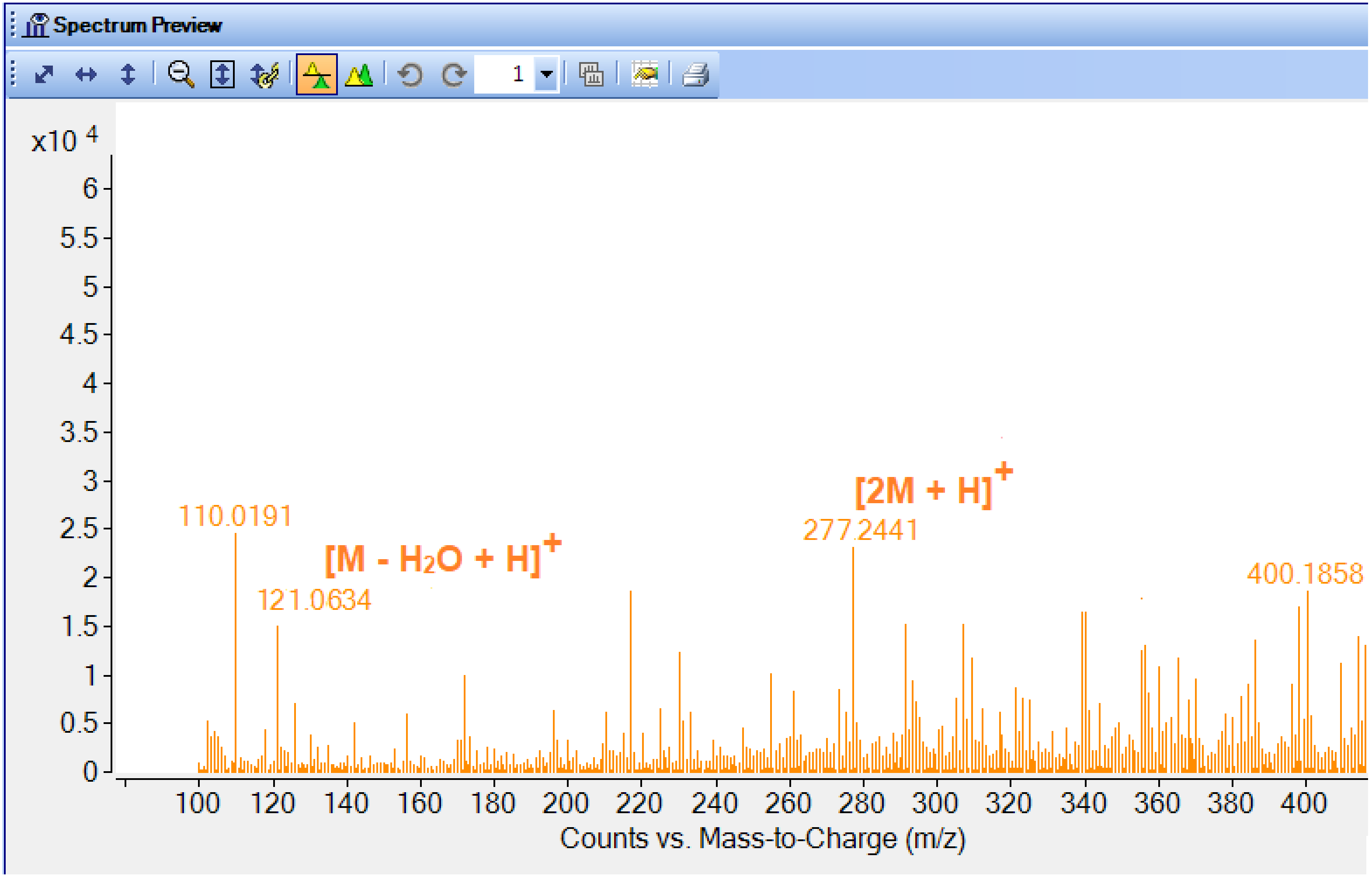

Supplementary Figure 13. ^{1}H NMR spectrum of ascosalitoxin (**7**) ($CDCl_3$, 400 MHz).

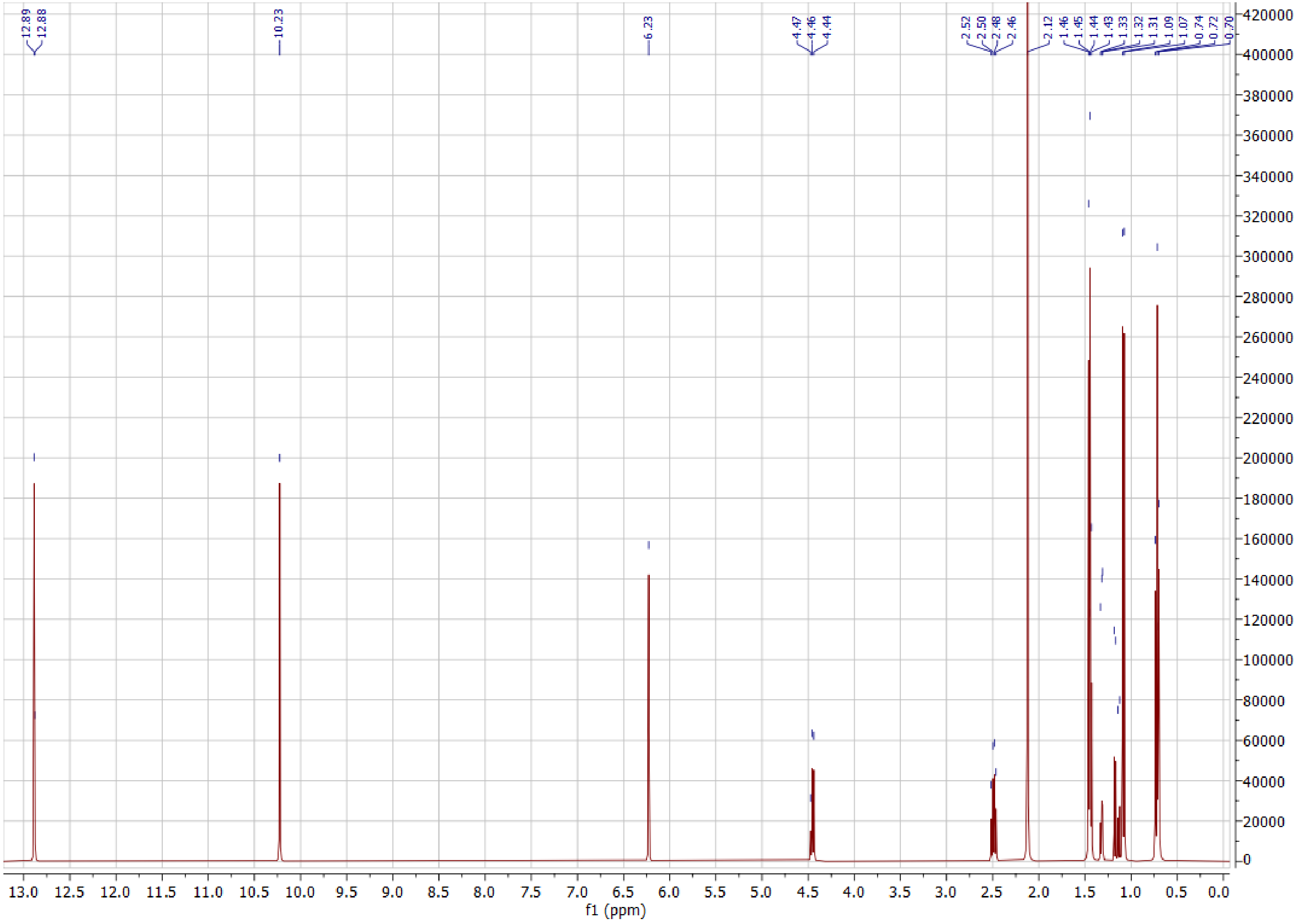

Supplementary Figure 14. ESI/MS (+) spectrum of ascosalitoxin (**7**).

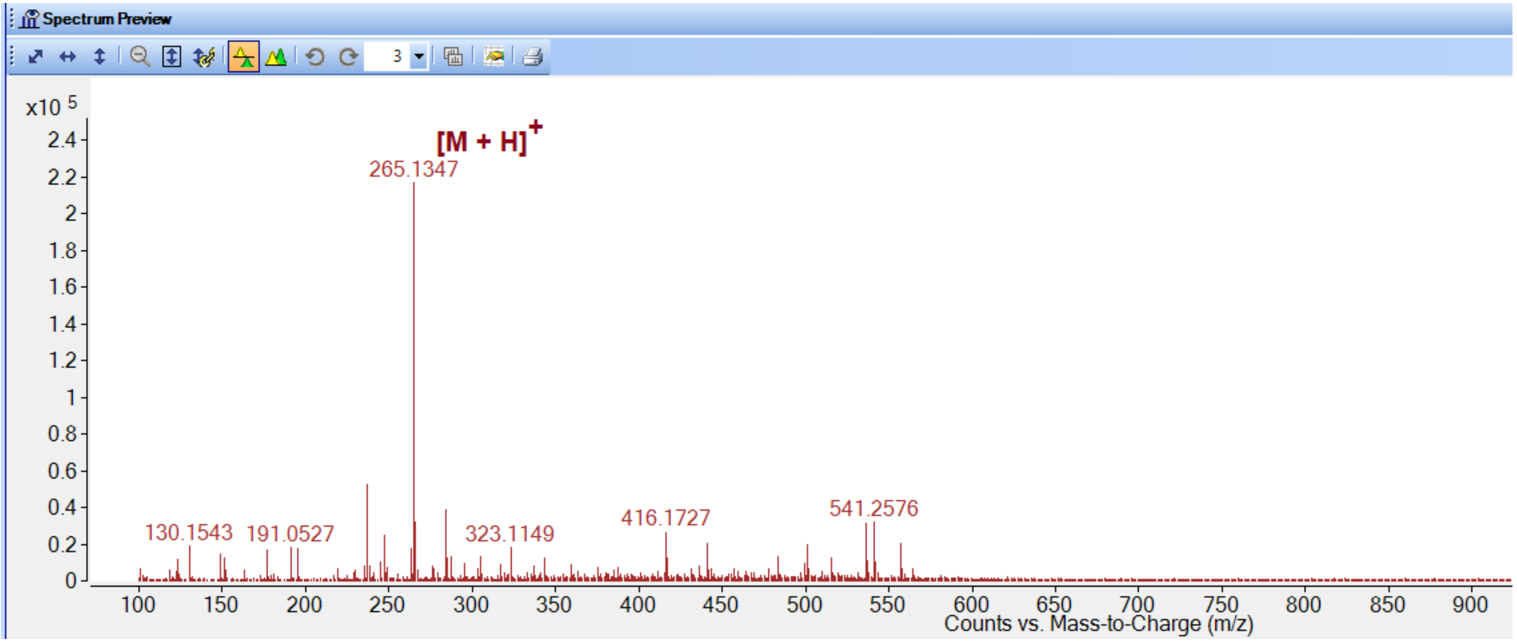

Supplementary Figure 15. Images of the symptoms of each of the compounds.

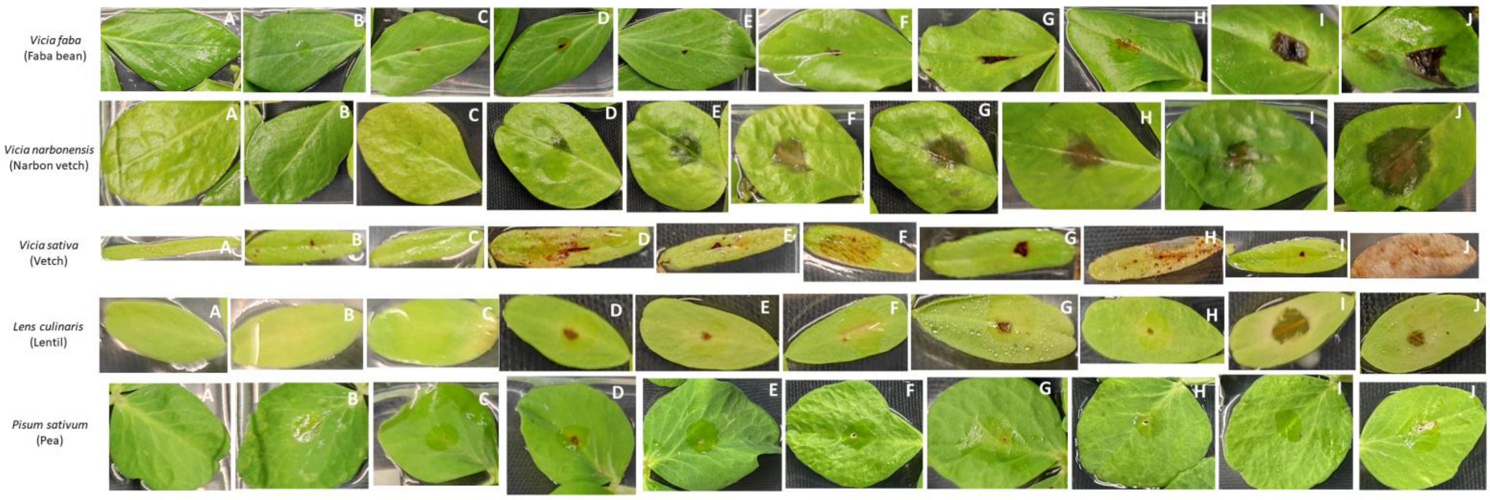

Figure 15. Symptoms developed on detached leaves of several legume hosts treated as follow: (a) uninoculated, (b) water, (c) methanol (MeOH 5%), (d) ascoclorin, (e) ascofuranol, (f) (*R*)-mevalonolactone, (g) ascosalipyrone, (h) benzoic acid, (i) tyrosol and (j) ascosalitoxin.

Supplementary Table 1. Diseased area (mm^2) measured on detached leaves of several legume crops with exudates from the fungus *Ascochyta fabae* growth in vitro on 3 different culture media (Czapek-Dox = CD, Potato Dextrose Broth = PDB and Rice) at concentrations of 0.5, 1 and 2 mg/mL. Negative (blank untreated, water and MeOH 5%) controls were also included. The experiment was repeated four times.

	Host plant									
**Treatment**	**Faba bean**		**Narbon vetch**		**Common vetch**		**Lentil**		**Pea**	
**Concentration 0.5 mg/mL**										
Blank	0.0±0.0	b	0.0±0.0	d	0.0±0.0	b	0.0±0.0	b	0.0±0.0	c
Water	0.0±0.0	b	2.9±1.6	d	0.2±0.1	b	0.0±0.0	b	0.3±0.2	c
MeOH	3.4±1.5	b	17.3±3.3	c	0.2±0.2	b	0.5±0.5	b	0.4±0.2	c
CD	45.2±11.1	a	65.3±17.1	a	25.6±4.7	a	16.1±8.5	a	16.1±3.6	a
PDB	63.8±29.1	a	53.5±8.9	a	16.8±9.2	a	0.0±0.0	b	9.9±6.5	b
Rice	61.7±16.3	a	36.9±4.7	b	3.9±3.4	b	0.0±0.0	b	7.8±2.6	b
**Concentration 1 mg/mL**										
Blank	0.0±0.0	c	0.0±0.0	d	0.0±0.0	b	0.0±0.0	b	0.0±0.0	c
Water	0.0±0.0	c	2.9±1.6	d	0.2±0.1	b	0.0±0.0	b	0.3±0.2	c
MeOH	3.4±1.5	c	17.3±3.3	c	0.2± 0.1	b	0.6± 0.5	b	0.4±0.2	c
CD	58.7±10.2	a	65.9±6.4	a	27.4±14.6	a	21.5±9.5	a	18.1±2.6	a
PDB	43.3±8.3	b	36.5±5.5	a	19.0±7.4	a	4.2±3.9	ab	8.1±4.4	b
Rice	82.3±24.5	a	47.6±19.3	b	30.1±10.4	a	16.9±7.5	a	12.3±4.4	b
**Concentration 2 mg/mL**										
Blank	0.0±0.0	b	0.0±0.0	b	0.0±0.0	c	0.0±0.0	c	0.0±0.0	b
Water	0.0±0.0	b	2.9±1.6	d	0.2±0.1	b	0.0±0.0	b	0.3±0.2	c
MeOH	3.4±1.5	c	17.3±3.3	c	0.2±0.2	b	0.5±0.4	b	0.4±0.3	c
CD	215.0±65.4	a	144.4±1.8	a	37.3±7.8	ab	20.6±7.8	a	80.7±57.4	a
PDB	157.0±25.33	a	43.9±7.9	b	44.2±12.9	a	1.6±0.8	bc	4.0±1.9	b
Rice	94.4±22.8	b	31.9±2.8	b	26.0±11.7	b	11.5±4.8	ab	9.2±3.4	b

Values, per column and treatment, followed by different letters differ significantly at $P < 0.01$.

Table 2. Diseased area (mm^2) measured in leaves detached from various legume crops with compounds produced by the exudate of the *Ascochyta fabae* fungus from the three-growth media at concentrations of 1, 10 and 100µM. Negative controls (untreated blank, water and MeOH 5%) were also included. *P*-value compared with value from MeOH 5% control.

Damage Area (mm^{-2})		*Vicia faba* (cv. Baraca)		*Vicia narbonensis* (cv. Saoret)		*Vicia sativa* (cv. Buzza)		*Lens culinaris* (cv. Pardina)		*Pisum sativum* (cv. Messire)	
		Mean	p value	Mean	p value	Mean	p value	Mean	p value	Mean	p value
Blank		1.0±0.1	-	0.7±0.01	-	2.9±0.7	-	0.1±0.1	-	0.3±0.1	-
Water		5.0±0.9	-	3.6±0.8	-	2.6±0.5	-	0.9±0.2	-	1.3±0.2	-
MeOH		6.0±1.1	-	3.8±0.9	-	3.3±0.7	-	1.3±0.3	-	1.2±0.2	-
Ascochlorin (**1**)	1µM	12.1±2.9	0.011*	17.8±3.5	<0.001*	5.6±2.5	0.154	1.7±0.2	0.394	1.7±0.4	0.157
	10µM	12.7±4.2	0.004*	16.0±4.8	<0.001*	1.4±0.3	0.183	1.0±0.2	0.409	1.7±0.4	0.169
	100µM	14.0±1.9	<0.001*	22.9±4.0	<0.001*	2.0±0.8	0.35	1.3±0.1	0.871	1.9±0.3	0.067
Ascofuranol (**2**)	1µM	4.7±1.4	0.508	16.0±5.9	<0.001*	0.9±0.1	0.253	1.6±0.3	0.554	1.4±0.2	0.598
	10µM	8.5±2.5	0.179	18.2±2.1	<0.001*	5.8±3.4	0.213	1.8±0.4	0.295	1.5±0.4	0.456
	100µM	10.8±2.7	0.011*	41.9±7.4	<0.001*†	8.3±4.5	0.019*	2.3±0.7	0.055	3.0±1.2	<0.001*†
(*R*)-mevalonolactone (**3**)	1µM	11.7±3.6	0.25	8.0±2.5	0.221	0.8±0.1	0.066	1.8±0.4	0.387	1.6±0.2	0.176
	10µM	12.9±2.5	0.148	10.1±4.3	0.067	1.2±0.2	0.112	1.6±0.5	0.552	1.6±0.3	0.255
	100µM	20.6±11.5	0.003*	30.5±9.7	<0.001*†	2.4±0.9	0.491	1.7±0.5	0.457	1.5±0.4	0.245
Ascosalipyrone (**4**)	1µM	9.9±4.1	0.229	5.3±2.6	0.696	1.8±1.0	0.237	2.3±0.6	0.073	1.8±0.4	0.272
	10µM	12.7±4.6	0.063	7.4±2.2	0.328	2.4±1.0	0.48	2.2±0.4	0.104	2.0±0.5	0.175
	100µM	18.4±6.5	<0.001*	46.1±11.9	<0.001*†	2.4±1.1	0.498	2.5±0.8	0.067	4.2±1.5	<0.001*†
Benzoic acid (**5**)	1µM	7.2±2.2	0.712	25.5±9.8	<0.001*	1.7±0.8	0.658	2.3±0.7	0.193	1.4±0.2	0.608
	10µM	11.8±3.6	0.002*	24.4±9.2	<0.001*	5.6±3.0	0.498	2.0±0.7	0.370	1.5±0.3	0.501
	100µM	16.6±6.3	0.003*	27.3±6.6	<0.001*	16.5±9.6	<0.001*†	4.0±1.8	<0.001*†	3.1±1.3	<0.001*†
Tyrosol (**6**)	1µM	18.5± 4.1	0.008*	27.2±5.3	<0.001*	2.2±0.8	0.415	1.3±0.4	0.945	1.1±0.2	0.653
	10µM	17.7±6.3	0.016*	24.2±6.4	<0.001*	1.7±0.5	0.209	1.6±0.3	0.786	0.8±0.1	0.177
	100µM	20.5±8.8	<0.001*	31.9±5.8	<0.001*	3.3±1.6	0.977	7.3±3.2	<0.001*†	0.9±0.1	0.413
Ascosalitoxin (**7**)	1µM	9.9±2.3	0.187	39.1±4.8	<0.001*	5.0±3.2	0.597	0.9±0.4	0.375	1.6±0.3	0.293
	10µM	29.8±6.4	<0.001*	40.6±5.1	<0.001*	3.9±1.4	0.829	1.3±0.5	0.882	1.2±0.1	0.856
	100µM	26.2±5.7	<0.001*†	36.5±15.9	<0.001*	20.0±7.8	<0.001*†	1.7±0.3	0.438	3.7±0.7	<0.001*†

Asterisk (*) indicates values significantly different from control MeOH 5%. Within each metabolite, the symbol (†) indicates significant differences between the concentrations tested.
